# DNA cleavage by CgII and NgoAVII requires interaction between N- and R-proteins and extensive nucleotide hydrolysis

**DOI:** 10.1093/nar/gku1236

**Published:** 2014-11-27

**Authors:** Mindaugas Zaremba, Paulius Toliusis, Rokas Grigaitis, Elena Manakova, Arunas Silanskas, Giedre Tamulaitiene, Mark D. Szczelkun, Virginijus Siksnys

**Affiliations:** 1 Department of Protein–DNA Interactions, Institute of Biotechnology, Vilnius University, Graiciuno 8, LT-02241 Vilnius, Lithuania; 2 DNA–Protein Interactions Unit, School of Biochemistry, Medical Sciences Building, University of Bristol, Bristol BS8 1TD, UK

## Abstract

The stress-sensitive restriction-modification (RM) system CglI from *Corynebacterium glutamicum* and the homologous NgoAVII RM system from *Neisseria gonorrhoeae* FA1090 are composed of three genes: a DNA methyltransferase (M.CglI and M.NgoAVII), a putative restriction endonuclease (R.CglI and R.NgoAVII, or R-proteins) and a predicted DEAD-family helicase/ATPase (N.CglI and N.NgoAVII or N-proteins). Here we report a biochemical characterization of the R- and N-proteins. Size-exclusion chromatography and SAXS experiments reveal that the isolated R.CglI, R.NgoAVII and N.CglI proteins form homodimers, while N.NgoAVII is a monomer in solution. Moreover, the R.CglI and N.CglI proteins assemble in a complex with R_2_N_2_ stoichiometry. Next, we show that N-proteins have ATPase activity that is dependent on double-stranded DNA and is stimulated by the R-proteins. Functional ATPase activity and extensive ATP hydrolysis (∼170 ATP/s/monomer) are required for site-specific DNA cleavage by R-proteins. We show that ATP-dependent DNA cleavage by R-proteins occurs at fixed positions (6–7 nucleotides) downstream of the asymmetric recognition sequence 5′-GCCGC-3′. Despite similarities to both Type I and II restriction endonucleases, the CglI and NgoAVII enzymes may employ a unique catalytic mechanism for DNA cleavage.

## INTRODUCTION

Restriction-modification (RM) systems protect bacterial cells against invasion by foreign DNA (e.g. phage or plasmid DNA). RM systems are composed of two principal enzymatic activities: an endonuclease and a methyltransferase. Restriction endonucleases (REases) cleave DNA that do not contain characteristic modifications (methyl groups) at defined sites within a specific recognition sequence. The host DNA is protected against nucleolytic attack because it is methylated within the recognition sequences by the cognate methyltransferase activity of the RM system. According to the genetic organization, subunit composition, cofactor requirement and mode of action, the RM systems were subdivided into four types: Type I, II, III or IV (1). Here we investigated a new class of RM system, exemplified by CglI and NgoAVII, which does not readily conform to the classical Types.

The CglI RM system from *Corynebacterium glutamicum* (ATCC 13032) is inactivated under various stress conditions (heat, organic solvents, pH shift or detergents), enabling the transfer of plasmid DNA from a donor (e.g. *Escherichia coli*) by either conjugation or phage infection (2,3). Inactivation of CglI may allow the host to more readily adapt to environmental changes by allowing acquisition of foreign genetic material (4). Due to its potential for inactivation, the CglI system was named as stress-sensitive restriction system. The CglI genes are located within the large prophage CGP3, which can be induced under SOS response conditions (5,6). It remains to be established whether prophage excision from the *Corynebacterium glutamicum* genome is linked to the inactivation of the CglI RM system under stress conditions (7).

**Figure 1.**
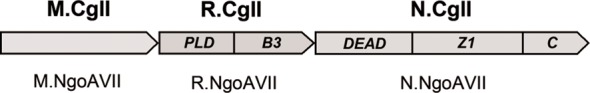
Organization of stress-sensitive restriction-modification systems from *Corynebacterium glutamicum* (CglI) and *Neisseria gonorrhoeae* FA1090 (NgoAVII). M.CglI (ORF NCgl1703) and M.NgoAVII (Ngo0365) are C5-methyl DNA methyltransferases. R.CglI (NCgl1704) and R.NgoAVII (Ngo0364) are putative REases with the PLD-superfamily nucleolytic and B3-like DNA binding domains. N.CglI (NCgl1705) and N.NgoAVII (Ngo0363) are predicted SF2 helicases/ATPases containing uncharacterized Z1-superfamily and C-terminal domains.

Previously the CglI system was assigned as Type II (REBASE, http://rebase.neb.com) (8). However, its genetic organization differs from typical Type II RM systems that are usually composed of only REases and methyltransferase genes (1). The CglI system is instead organized as three genes: a methyltransferase (M.CglI), a putative PLD-family endonuclease (R.CglI) and a predicted DEAD-family helicase/adenosine triphosphatase (ATPase) (N.CglI) (Figure 1). The NgoAVII RM system, identified by bioinformatics in *Neisseria gonorrhoeae* FA1090 (ATCC 700825) was also assigned as a stress-sensitive restriction system (BLAST), and correspondingly is composed of three genes homologous to the CglI RM system: M.NgoAVII, R.NgoAVII and N.NgoAVII, respectively (Figure 1, Supplementary Figures S1, S2 and S3). Given the unusual stress response and presence of a putative ATPase component, the CglI and NgoAVII RM systems may use a new, uncharacterized mechanism for DNA cleavage.

The putative REases R.CglI and R.NgoAVII contain predicted PLD-superfamily HXK nuclease active site motifs within their N-terminal domains and C-terminal B3-type DNA-binding domains (Figure 1, Supplementary Figure S2) (see the accompanying paper) (9). An N-terminal PLD-superfamily domain is also characteristic of the well-studied Type IIS REase BfiI (recognition sequence 5′-ACTGGG-3′), in which two N-terminal nucleolytic domains dimerize to form a single active site that acts sequentially to make double-strand DNA breaks at fixed positions downstream of the recognition sequence (10,11). Although the R.CglI and R.NgoAVII proteins hydrolyzed the artificial substrate bis(*p*-nitrophenyl) phosphate, in contrast to BfiI (12), they showed no DNA cleavage activity (Stonyte, Toliusis and Siksnys, unpublished data) most likely because the third protein (N.CglI or N.NgoAVII, respectively) is also required. A necessity for cooperation between R and N protein can also be inferred based on *in vivo* phage-resistance and conjugation experiments using CglI (3,13) and from *in vitro* DNA cleavage experiments with the homologous BceSIV RM system from *Bacillus cereus* (7).

*In silico* amino acid sequence analysis predicted that N.CglI and N.NgoAVII are composed of three domains: an N-terminal DEAD-superfamily helicase domain (Superfamily 2, SF2); a domain similar to the Z1-superfamily; and a distinct C-terminal domain (Figure 1, Supplementary Figure S3). The uncharacterized Z1 domain is often found associated with a SF2 helicase domain (14). The N-proteins possess the characteristic ATP- and Mg^2+^-binding amino acid sequence motifs, consistent with ATPase activity (Supplementary Figure S3). Similar DEAD domains are found in SF2 helicases involved in ATP-dependent RNA or DNA unwinding/translocation (15). However, the N-protein helicase motifs are only distantly-related to those characteristic of Type I and Type III RM enzymes (data not shown).

The M.CglI and M.NgoAVII proteins have high amino acid sequence similarity (62% identity and 76% similarity) and are Type II 5-methylcytosine methyltransferases that recognize the 5′-GCSGC-3′ DNA sequence (where S represents either G or C) (Supplementary Figure S1) (16,17). Like typical Type II methyltransferases, they are active as isolated proteins (Supplementary Figure S5).

In this paper, we report a biochemical characterization of the putative REases R.CglI and R.NgoAVII (also referred to as ‘R-proteins’) and cognate ATPases N.CglI and N.NgoAVII (also referred to as ‘N-proteins’). Following recombinant gene expression in *E. coli*, we purified individual proteins and their domains to apparent homogeneity. Using co-expression of R.CglI and N.CglI, the RN.CglI complex with an R_2_N_2_ stoichiometry was also isolated. We demonstrate that both R.CglI and R.NgoAVII are endonucleases that require ATP hydrolysis by the corresponding N-proteins to produce site-specific DNA cleavage at fixed positions downstream of the asymmetric recognition sequence 5’-GCCGC-3’. On the basis of their activities, we propose that CglI and NgoAVII represent a new sub-class of RM enzyme with characteristics of both Type I and II RM systems.

## MATERIALS AND METHODS

### Expression and purification of CglI and NgoAVII proteins

*Corynebacterium glutamicum* ATCC 13032 and *Neisseria gonorrhoeae* FA1090 genomic DNA (both from LGC Standards) were used to produce recombinant genes of R.CglI/N.CglI, R.NgoAVII/N.NgoAVII or DNA fragments corresponding to individual domains that later were ligated into expression and co-expression vectors (Supplementary Tables S1 and S2). Complete gene sequences were confirmed. His_6_- or StrepII-tagged proteins were expressed and purified using liquid chromatography as described in Supplementary Tables S1 and S2. The homogeneity of the protein preparations (>90%) was determined using sodium dodecyl sulphate-polyacrylamide gel electrophoresis (SDS-PAGE) and gel densitometry. The identity of the purified proteins was confirmed by mass spectrometry (data not shown). Protein concentrations were determined by measuring absorbance at 280 nm using appropriate extinction coefficient (Supplementary Tables S1 and S2).

### Mutagenesis

The R.CglI (H105A), N.CglI (D158A+E159A), R.NgoAVII (H104A) and N.NgoAVII (D150A+E151A) mutants were produced by QuikChange Site-Directed Mutagenesis (18). Sequencing of the entire gene for each mutant confirmed that only the designed mutations had been introduced.

### Gel filtration

Gel filtration was carried out at room temperature on an AKTA Avant system using a Superdex 200 HR column (GE) pre-equilibrated with 20 mM Tris–HCl (pH 8.0 at 25°C), 300 mM NaCl and 5 mM 2-mercaptoethanol. Protein samples at 20 μM (monomer) loading concentration were prepared in 50 μl of the above buffer. Elution from the column was monitored by measuring absorbance at 280 nm. The apparent molecular weights of proteins were evaluated from the elution volume using a series of standards (Gel Filtration Calibration Kit from GE Healthcare).

### Small angle X-ray scattering (SAXS) experiments

The wild-type (wt) RN.CglI complex was dialyzed against 20 mM Tris–HCl (pH 8.0 at 25°C), 400 mM KCl, 0.1 mM ethylenediaminetetraacetic acid (EDTA) and 1 mM dithiothreitol (DTT). R.CglI, R.NgoAVII, N.CglI and N.NgoAVII were transferred into the same buffer using desalting NAP columns (GE Healthcare). Proteins were concentrated using centrifugal concentrators (Amicon Ultra-0.5, Millipore). All samples were processed at 15 000 *g* for 15–20 min.

Small angle X-ray scattering (SAXS) data were collected at P12 EMBL beam-line at PETRAIII storage ring of the DESY synchrotron in Hamburg (Germany). The sample-to-detector (Pilatus 2M) distance used in the measurements was 3.1 m. Data were collected for the momentum transfer *s* (*s* = 4*π* sin(*θ*)/*λ*, where 2*θ* is a scattering angle and *λ* is x-ray wavelength, 0.124 nm) range from 0.07579 to 4.66533 nm^−1^, except the R.CglI samples, which were measured in *s* range from 0.02906420 to 4.491070 nm^−1^. Data were truncated at the first point of the Guinier region, as calculated by AUTORG (19). Real space *R*_g_ and *I*(0) were calculated using GNOM (20). *D*_max_ was estimated manually by interactive GNOM runs and compared with the value calculated by DATGNOM (19). Unprocessed scattering data */*(s) with subtracted buffer scattering, Kratky plots and distance distribution functions P(r) of protein samples are presented in Supplementary Figure S4. Structural parameters derived from the SAXS measurements are presented in Supplementary Table S3.

Molecular weight estimations of individual proteins and the corresponding complexes are presented in Table 1. Molecular weights were calculated from the Fourier-transformed scattering data by the method described in (21) using the MoW program run on the server (http://www.if.sc.usp.br/∼saxs/saxsmow.html).

**Table 1.**
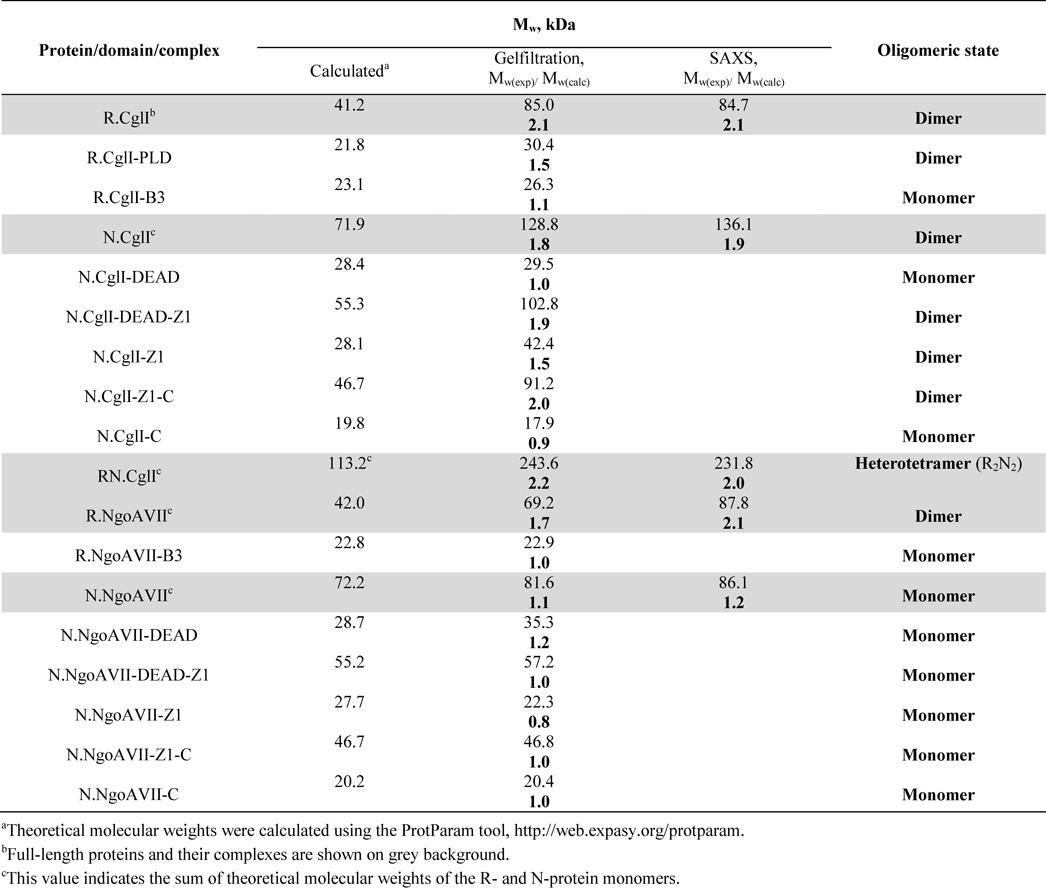
Oligomeric state of the CglI and NgoAVII proteins and their domains

### Gel mobility-shift assay

DNA binding by the CglI and NgoAVII proteins was analyzed by the electrophoretic mobility shift assay (EMSA) using a 33 bp cognate duplex (5′-TACAATCGATAATAGCCGCCGTGCGAGCCCATT-3′/3′-ATGTTAGCTATTATCGGCGGCACGCTCGGGTAA-5′, the recognition sequence is underlined) or a 33 bp non-cognate DNA duplex (5′-TACAATCGATAATACCAGGCGTGCGAGCCCATT-3′/3′-ATGTTAGCTATTATGGTCCGCACGCTCGGGTAA-5′). DNA (final concentration 1 nM) was incubated with proteins (final concentrations from 1 to 500 nM monomer) for 15 min in 20 μl of 40 mM Tris-acetate (pH 8.3 at 25°C), 0.1 mM EDTA, 0.1 mg/ml bovine serum albumin (BSA) and 10% (v/v) glycerol at room temperature. Free DNA and protein–DNA complexes were separated by electrophoresis using 8% (w/v) acrylamide gels (29:1 acrylamide/bisacrylamide in 40 mM Tris-acetate, pH 8.3 at 25°C and 0.1 mM EDTA). In the case of the NgoAVII proteins, EDTA in the binding buffer and loading gels was replaced by 5 mM Ca-acetate. Samples were separated at room temperature for 3 h at ∼6 V/cm. Radiolabeled DNA was detected and quantified using the Cyclone phosphorimager and the OptiQuant software (Packard Instrument). Apparent *K*_d_ values for DNA binding were determined as described in (22).

### ATPase assay

ATPase reactions were performed at 37°C for 1 h in 33 mM Tris-acetate (pH 7.0 at 25°C), 0.1 mg/ml BSA, 10 mM Mg-acetate and 150 mM K-acetate (only for CglI proteins), supplemented with 50 μM [α^32^P]ATP (Hartmann Analytic, Braunschweig, Germany) and 0.01 μg/μl of various DNAs (as indicated): (i) phage λ DNA (dam^+^dcm^+^), (ii) pBR322, (iii) pBR322 methylated at the CglI/NgoAVII recognition sites, (iv) cognate fragment, (v) noncognate fragment, (vi) cognate oligoduplex (5′-GATCGAGCCGCCTACG-3′/3′-CTAGCTCGGCGGATGC-5′, the recognition sequence is underlined), (vii) non-cognate oligoduplex (5′-GATCGACCAGGCTACG-3′/3′-CTAGCTGGTCCGATGC-5′) and (viii) single-stranded M13mp18 (Supplementary Data). Reactions were initiated by adding the CglI (40 nM) or NgoAVII (40 or 100 nM) proteins and were stopped by addition of EDTA to 30 mM. An aliquot (1 μl) was spotted onto a polyethyleneimine-cellulose thin-layer plate, the substrate ATP and product ADP separated by chromatography in a 0.325 M KH_2_PO_4_ (pH 3.5 at 25°C) and the plates visualized using Fujifilm FLA-5100 fluorescent image analyzer (Fujilm, Tokyo, Japan).

Malachite green assays (BioAssay Systems) were used to measure the ATP hydrolysis rate by detection of liberated free phosphate. Reactions were performed at 37°C in the same reaction buffer as in the radioactivity assay, and containing 0.02 μg/μl phage λ DNA, ATP (1, 2, 4 or 6 mM), 1 mM (d)NTP and 2.5 or 10 mM divalent metal ions (Mg^2+^, Mn^2+^, Ca^2+^ or Ni^2+^) for NgoAVII and CglI, respectively. Reactions were initiated by adding N.CglI or N.NgoAVII (10 nM) plus R.CglI or R.NgoAVII (200 nM). Aliquots were removed at fixed time intervals and the reactions stopped by adding EDTA to 9.5 mM. The relationship between the absorbance and phosphate concentration was established by using KH_2_PO_4_ as a standard. ATPase rates are quoted as the mean of three independent experiments.

### DNA cleavage assay

DNA cleavage reactions were performed for 1 h at 37°C in reaction buffer: 33 mM Tris-acetate (pH 7.0 at 25°C); 0.1 mg/ml BSA; either 50 mM (for NgoAVII) or 150 mM (for CglI) K-acetate; either 2.5 mM (for NgoAVII) or 10 mM (for CglI) divalent metal ions (Mg^2+^, Mn^2+^ or Ca^2+^); 1 mM DTT; 2 or 4 mM adenylyl-imidodiphosphate (AMP-PNP) or ADP; 2 or 4 mM (d)NTP; 25 mM phosphocreatine; and 5 U/ml creatine kinase. The reactions were supplemented with 0.01 μg/μl of various dsDNAs (as indicated): (i) phage λ DNA (dam^+^dcm^+^), (ii) pBR322, (iii) pBR322 methylated at the CglI/NgoAVII recognition sites, (iv) pUC19 or (v) 0-site plasmid (without CglI/NgoAVII recognition sequences and generated from pMDS36a by the site-directed mutagenesis (23)). Reactions were initiated by adding N.CglI (200 nM) plus R.CglI (200 nM) or N.NgoAVII (100 or 200 nM) plus R.NgoAVII (100 or 200 nM) to the reaction mixture. Reactions were stopped by addition of 1:1 phenol–chloroform. The aqueous fraction was mixed with loading solution (75 mM EDTA, 0.025% (w/v) bromphenol blue, 50% (v/v) glycerol, 0.3% (w/v) SDS, pH 8.0 at 25°C) and incubated at 70°C for 15 min. The products were separated by electrophoresis through 0.8 or 1.0% (w/v) agarose gels and visualized under ultraviolet light following ethidium bromide staining.

To analyze the DNA cleavage position of CglI and NgoAVII, the 1-site plasmid (10 or 2 nM, respectively), generated from pMDS36a by the site-directed mutagenesis (23), was incubated with N.CglI (500 nM) plus R.CglI (500 nM) or N.NgoAVII (100 nM) plus R.NgoAVII (100 nM) for 1 h at 37°C in reaction buffer. Linearized plasmids were purified following agarose gel electrophoresis using the GeneJET Gel Extraction Kit (Thermo Fisher Scientific, Vilnius). The linear purified DNA was sequenced directly with the primers 5′-GAACTCTGTAGCACCGCC-3′ [top strand] and 5′-CACTCAAAGGCGGTAATACGG-3′ [bottom strand].

## RESULTS

### Expression and purification of proteins

The R- and N-proteins and their domains were expressed with either His_6_- or StrepII-tags (see ‘Materials and Methods’ section, Supplementary Tables S1 and S2). A set of alanine-substitution mutations of the putative active sites within the R- and N-proteins was generated: H105 (R.CglI) and H104 (R.NgoAVII) belonging to the predicted HXK active site motif of the PLD-superfamily nucleolytic domain; and D158+E159 (N.CglI), and D150+E151 (N.NgoAVII) located in the DEAD domain and involved in Mg^2+^ coordination (Supplementary Figures S2 and S3). Also, both R- and N-proteins from either system were co-expressed using a single recombinant vector (see ‘Materials and Methods’ section, Supplementary Table S1). All proteins were expressed in *E. coli* and were purified to an apparent homogeneity (>90%) by liquid chromatography. The co-expressed R.CglI (containing a StrepII-tag) and N.CglI (containing a His_6_-tag) formed the RN.CglI complex that was purified using serial affinity chromatographic columns (HisTrap HP and StrepTrap HP) (Supplementary Table S1). In the case of the co-expressed R.NgoAVII and N.NgoAVII proteins, a complex was not obtained (data not shown). The identity of all purified proteins was confirmed by mass spectrometry (data not shown).

### Oligomeric assembly

The oligomeric assembly of the individual R- and N-proteins and their domains was analyzed in solution by gel filtration (Table 1, Supplementary Figures S6 and S7). Both R.CglI and its isolated PLD domain eluted from the column as dimers, while the isolated R.CglI-B3 domain eluted as a monomer (Supplementary Figure S6, Table 1). N.CglI, its isolated DEAD-Z1 domain, its isolated Z1 domain and its isolated Z1-C domain all eluted as dimers, while the isolated DEAD and C-domains eluted as monomers (Supplementary Figure S6, Table 1). Full-length R.NgoAVII also eluted as a dimer, while the R.NgoAVII-B3 domain eluted as a monomer (Supplementary Figure S7, Table 1). N.NgoAVII and all its isolated domains eluted as monomers (Supplementary Figure S7, Table 1). SAXS experiments confirmed the same oligomeric state for the full-length R- and N-proteins as determined by gel filtration (Supplementary Figure S4, Table 1).

The molecular weight of the RN.CglI complex, obtained by co-expression of both the R.CglI and N.CglI proteins, was determined by gel filtration and SAXS to be close to the sum of molecular weights of the R.CglI and N.CglI dimers (Table 1, Supplementary Figures S4 and S6). Therefore, the R.CglI and N.CglI dimers assemble into a protein complex with a R_2_N_2_ stoichiometry. In addition, the stoichiometry of the RN.CglI complex was examined using densitometric analysis of the purified complex following SDS/PAGE electrophoresis (Supplementary Figure S8). The calculated molar ratio of the R.CglI and N.CglI proteins within the complex was 1:0.75. Since both proteins are dimers in solution, this ratio supports the R_2_N_2_ stoichiometry. The R_2_N_2_ CglI complex was also obtained by mixing the individual R.CglI and N.CglI proteins *in vitro* (data not shown). Higher-order complex formation was also observed upon mixing of the full-length R.CglI protein with either the N.CglI-DEAD domain or the N.CglI-DEAD-Z1 domain (Supplementary Figure S9). In contrast, a higher-order complex was not obtained if the PLD domain or B3 domain of R.CglI were mixed with the corresponding domains of N.CglI (data not shown). As with the co-expression study, a stable complex between the R.NgoAVII and N.NgoAVII was not observed upon mixing the proteins *in vitro* (Supplementary Figure S7, data not shown).

### DNA binding studies

**Figure 2.**
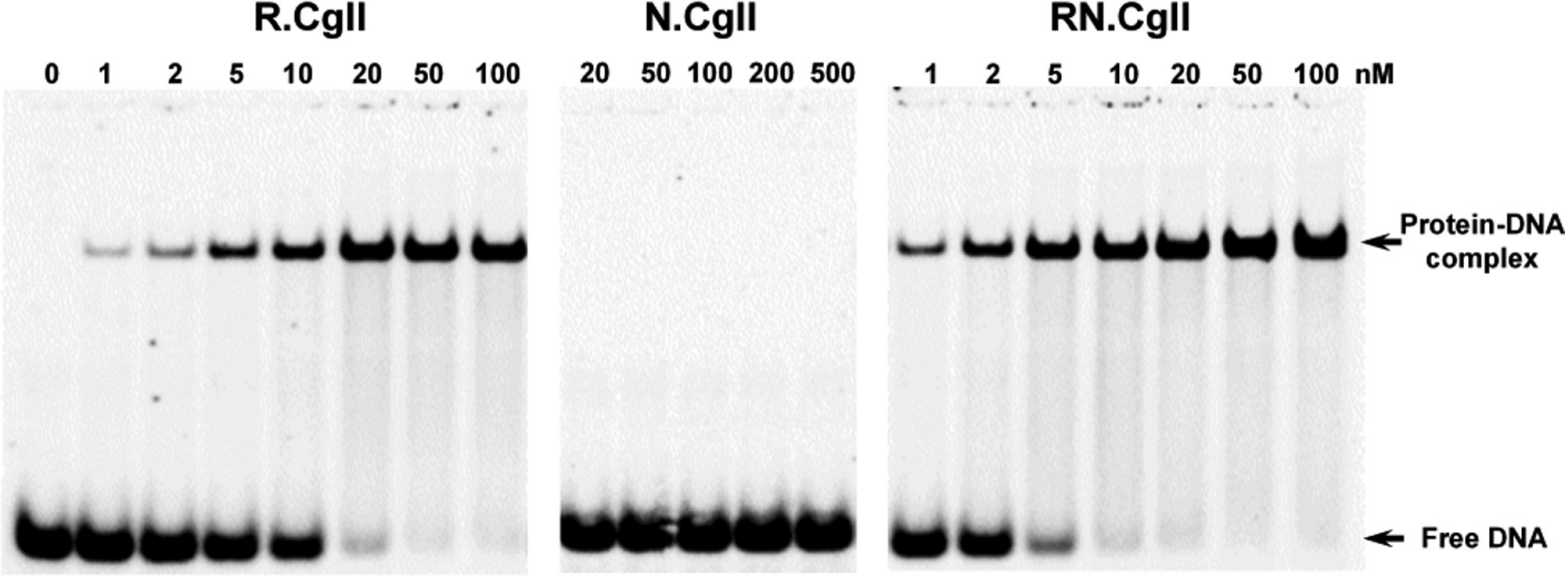
Cognate DNA binding by CglI proteins. The reactions contained 1 nM of the ^33^P-labeled cognate oligoduplex and the protein at the concentrations indicated above each lane. After 15 min at room temperature, the samples were subjected to native PAGE for 3 h and analyzed as described in ‘Materials and Methods’ section. The calculated *K*_d_ values are 9.2 ± 2.8 nM for R.CglI and 1.9 ± 0.6 nM for RN.CglI.

DNA binding by the CglI and NgoAVII proteins was studied by EMSA using ^33^P-labeled cognate and non-cognate oligoduplexes (Figure 2, Supplementary Figures S10, S11 and S12). In the case of the non-cognate oligoduplex, little or no DNA binding was observed with the R- or N-proteins, or with the RN-complex (Supplementary Figures S10 and S11). The R-proteins and their corresponding isolated B3 domains formed a specific complex with cognate DNA, while the N-proteins did not (Figures 2, Supplementary Figures S10, S11 and S12). The complete RN.CglI complex bound the cognate oligoduplex into a protein–DNA complex with the same electrophoretic mobility as the DNA complex formed by the isolated R.CglI protein (Figure 2). We rationalize these results as showing that the RN.CglI complex dissociates at the nanomolar protein concentrations used in the EMSA assay (as compared to the micromolar protein concentrations used in the gel filtration and SAXS assays). The observed complex is therefore between the dissociated R-protein and the DNA. On the basis of the EMSAs we propose that the R-proteins, and in particular the B3 domains, are responsible for the interaction with the DNA recognition sequence.

### ATP hydrolysis studies

**Figure 3.**
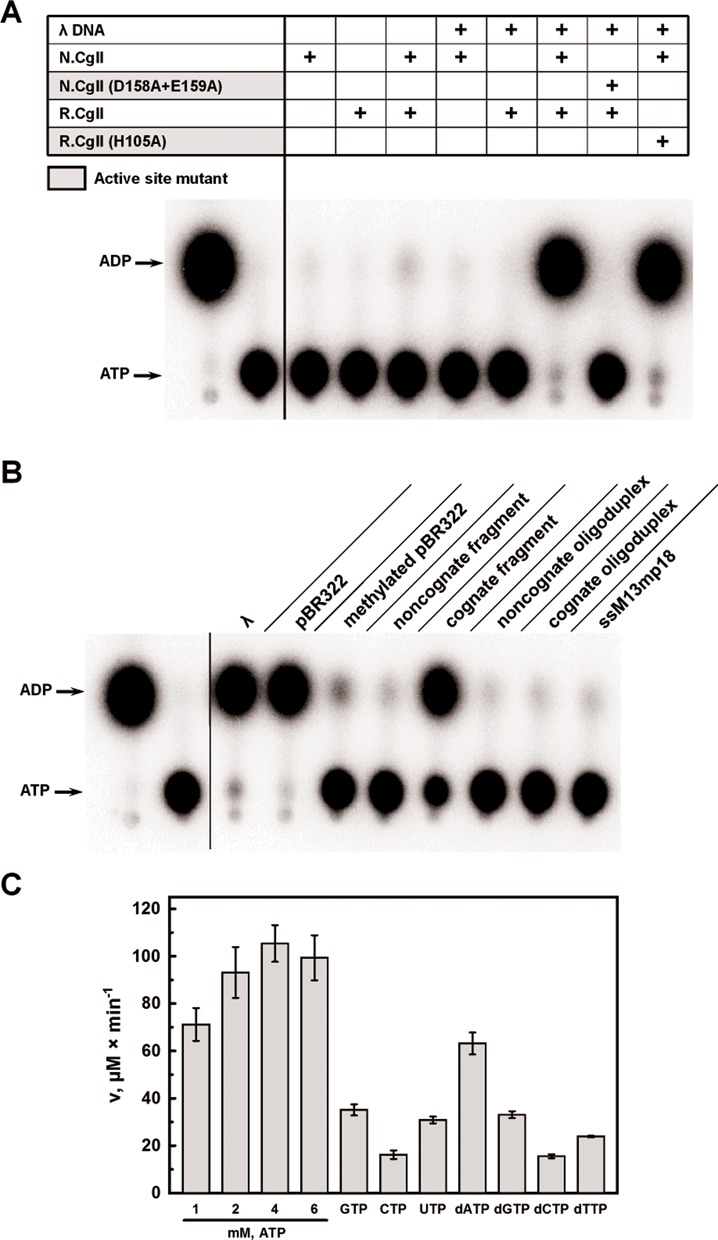
N.CglI ATPase activity. (**A**) Radioactive ATPase assay. ATPase reactions contained 50 μM [α^32^P]ATP, 0.01 μg/μl phage λ DNA, 40 nM N.CglI or R.CglI and were conducted as described in ‘Materials and Methods’ section. Reaction products were separated using thin-layer chromatography and visualized using a phosphoimager. (**B**) Dependence of CglI ATPase activity on different DNAs. Reactions were performed as in (A) using 0.01 μg/μl DNA concentration (see details in the text). (**C**) NTP hydrolysis rates. Reactions contained 0.02 μg/μl phage λ DNA, 10 nM N.CglI, 200 nM R.CglI, 1–6 mM ATP or 1 mM (d)NTP, as indicated, and were conducted as described in ‘Materials and Methods’ section. The malachite green assay was used to measure ATP hydrolysis through the detection of liberated free phosphate.

The presence of the characteristic SF2 helicase/ATPase motifs in the primary sequence of the N-proteins predicts that the proteins would be DNA-dependent ATPases (Supplementary Figure S3). To test this, the ATPase activity of the N-proteins was first analyzed by thin-layer chromatography using radioactive [α^32^P]ATP as a substrate (Figure 3A and Supplementary Figure S9A). In the presence of the N-proteins alone, ATPase activity was not detected, independent of the presence of DNA. Residual ATP hydrolysis was observed with N.NgoAVII and double-stranded bacteriophage λ DNA (Supplementary Figure S13A). However, the double-stranded DNA-dependent ATPase activity of the N-proteins increased significantly in the presence of the corresponding R-proteins (Figure 3A). The active site mutants of N.CglI (D158A+E159A) and N.NgoAVII (D150A+E151A) did not have any detectable ATP hydrolysis activity. In contrast, mutations in the N-terminal PLD domains of R.CglI (H105A) and R.NgoAVII (H104A) had no effect on the ATPase activity of the respective RN-complexes. These results confirm that the N-proteins are ATPases, which require both DNA and a catalytically active or inactive (see below) endonuclease subunit for maximal activity.

The ATPase activity of the RN-complexes was further analyzed in the presence of: various circular or linear double-stranded DNA substrates (see ‘Materials and Methods’ section and Supplementary Data), either lacking or containing different numbers of recognition sites (unmodified or modified by M.NgoAVII where stated); or circular single-stranded M13mp18 (Figures 3B and Supplementary Figure S13B). The RN.CglI complex showed ATPase activity in the presence of linear phage λ DNA, circular pBR322 plasmid or a linear DNA fragment containing a single recognition site (Figure 3B). Interestingly, a 16 bp cognate oligoduplex containing the CglI recognition sequence did not support ATP hydrolysis. Little or no ATP hydrolysis was observed in reactions containing pBR322 with methylated recognition sites, with a non-cognate DNA fragment, with a non-cognate oligoduplex, or with single-stranded DNA (Figure 3B). Similar results were obtained with N.NgoAVII, except that more pronounced ATP hydrolysis was observed with methylated pBR322 and with the non-cognate DNA (Supplementary Figure S13B). Thus, ATPase activity of the R_2_N_2_ complex is only supported by double-stranded DNA containing one or more unmodified recognition sites and with a length >16 bp.

In order to test the nucleotide triphosphate specificity of the RN-complexes, we compared the catalytic activity using either ribo- and deoxyribonucleotide cofactors (Figure 3C and Supplementary Figure S13C). For these experiments the malachite green assay was used to measure (d)NTP hydrolysis through detection of liberated free phosphate. The RN-complexes had a partial preference for ATP and dATP, but could nonetheless hydrolyze both GTP and dGTP efficiently (∼2-fold slower compared to ATP). Other dNTPs/NTPs could be utilized but with a lower hydrolysis rate (up to 5-fold slower compared to ATP) (Figure 3C and Supplementary Figure S13C). According to the hydrolysis rate measured at saturating ATP concentration (4 mM) and optimal concentrations of DNA and R.CglI, one molecule of N.CglI hydrolyses ∼176 ± 13 ATP/s whilst N.NgoAVII hydrolyses ∼174 ± 25 ATP/s.

We also studied the divalent metal ion dependence on the ATPase activity. The highest ATP hydrolysis rate was observed with Mg^2+^, whilst a lower rate (up to 2.5-fold in comparison to Mg^2+^) was determined with Mn^2+^ or Ca^2+^ (Supplementary Figure S14). No significant ATPase activity was detected in reactions containing EDTA (Supplementary Figure S14).

### DNA cleavage studies

**Figure 4.**
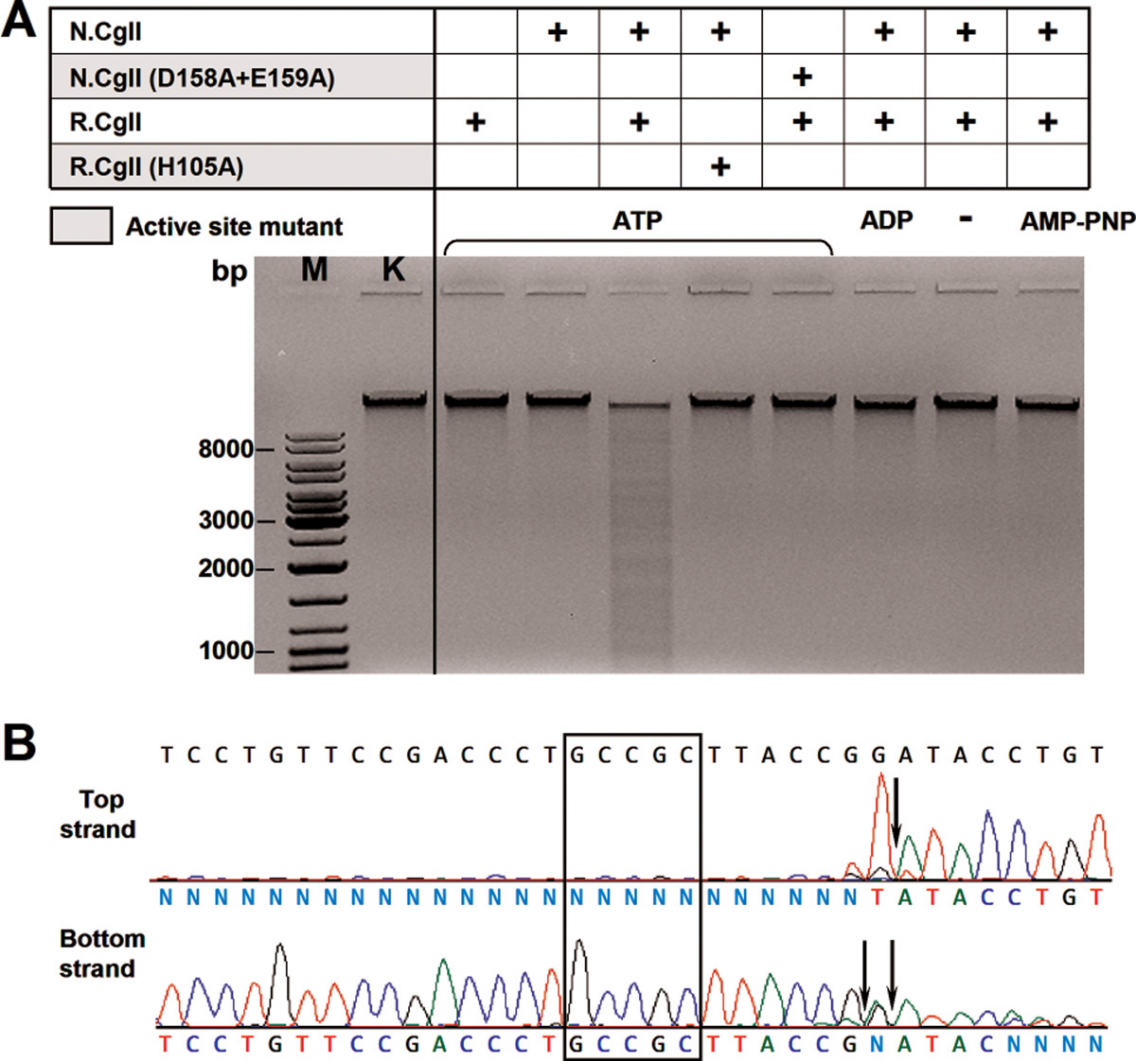
R.CglI nuclease activity. (**A**) Phage λ DNA cleavage by R.CglI. Reactions contained 0.01 μg/μl phage λ DNA, 200 nM R.CglI or N.CglI, 4 mM ATP, ADP or AMP-PNP (as indicated above the lanes) and were conducted as described in ‘Materials and Methods’ section. (**B**) Run-off sequencing to determine the cleavage position of R.CglI. The recognition sequence 5′-GCCGC-3′ is indicated by the rectangle whilst the cleavage sites are indicated by arrows.

DNA cleavage by the R-proteins was investigated using linear phage λ DNA as a substrate (which contains 181 CglI/NgoAVII recognition sites) (Figure 4 and Supplementary Figure S15A). The R-proteins alone did not cleave the DNA to any detectable extent. Reactions with supercoiled pBR322 (which contains 21 recognition sites) did not produce any detectable increase in nicked DNA, indicating that the isolated R-proteins cannot introduce a single-stranded DNA break (data not shown). However, both phage λ DNA (Figure 4) and pBR322 (data not shown) were fragmented by the R-proteins in the presence of their cognate N-proteins and ATP. Active site mutations either within the nucleolytic PLD-superfamily domain of the R-protein or within the SF2-helicase domain of the N-protein abolished DNA cleavage. DNA fragmentation was not observed in the absence of ATP or in the presence of ADP or AMP-PNP (a non-hydrolyzable analogue of ATP), suggesting that ATP hydrolysis is a prerequisite for cleavage. pBR322 with methylated recognition sites or a 0-site plasmid were not cleaved, as expected (data not shown). All divalent metal ions tested (Mg^2+^, Mn^2+^ and Ca^2+^) supported DNA cleavage, whereas DNA hydrolysis was not observed in the presence of EDTA which prevents ATP hydrolysis (data not shown). DNA cleavage was also observed in the presence of other (d)NTPs (data not shown). Thus, the catalytically active ATPases (N-proteins) and ATP hydrolysis are required for the DNA cleavage by the R-proteins.

In order to determine the cleavage position of the CglI and NgoAVII endonucleases, the DNA fragments obtained after ATP-dependent cleavage were analyzed using run-off sequencing (Figure 4B and Supplementary Figure S15B). In the case of CglI, DNA cleavage occurred 7 and 6/7 nucleotides downstream of the 5′-GCCGC-3′ site on the top and bottom strands, respectively. Similar results were obtained for NgoAVII.

## DISCUSSION

The stress-sensitive RM systems CglI (from *Corynebacterium glutamicum*) and NgoAVII (from *Neisseria gonorrhoeae* FA1090) were originally assigned as Type II RM systems. Here, we overproduced, purified and characterized *in vitro* the wt and mutant R- and N-proteins and their isolated domains from both systems. Our results show that, in contrast to other Type II enzymes, CglI and NgoAVII possess three enzyme activities: namely, a methyltransferase, an endonuclease and an ATPase, the last two of which are coupled (Figure 1).

### ATP hydrolysis by the N-proteins

The N-terminal domains of the N-proteins carry ATPase motifs characteristic of the helicases of the DEAD-superfamily (Supplementary Figure S1) (15). Helicases use ATP hydrolysis to unwind and/or translocate nucleic acids, and/or to remodel nucleic acids or nucleoprotein complexes (15,24,25). The Type I and III RM enzymes both contain domains with SF2 helicase motifs and require ATP hydrolysis for DNA cleavage (see below) (26). However, the DEAD domains within the N-proteins: have helicase motifs distinct to those found in Type I or III enzymes; are associated with uncharacterized Z1 domains that are not found associated with the helicase domains in Type I or III enzymes (14); and do not have the nuclease domain fused within a single polypeptide as seen in the HsdR subunits of Type I enzymes and Res subunits of Type III enzymes (27–29). We demonstrate here that the N-proteins do have ATPase activity on longer DNA (>16 bp) with non-methylated recognition sequences 5′-GCSGC-3′, but only when in complex with the cognate R-protein (Figure 3A and 3B, Supplementary Figures S13A and S13B). Both N.CglI and N.NgoAVII, like other DEAD-superfamily helicases, required divalent metal ions (Mg^2+^, Mn^2+^ or Ca^2+^) for ATPase activity and were inactive in the presence of EDTA (Supplementary Figure S14) (30). Mutations of the conserved amino-acid residues responsible for Mg^2+^ coordination (mutations D158A+E159A in N.CglI and D150A+E151A in N.NgoAVII) abolished ATP hydrolysis and cleavage (Figure 3A and Supplementary Figure S13A). However, active site mutations in R.CglI (H105A) or R.NgoAVII (H104A), did not affect the ATPase activity of the cognate N-proteins. The N-proteins showed weak preference for ATP and dATP, although, other deoxy- and ribonucleotides were also hydrolyzed (Figure 3C and Supplementary Figure S13C).

Our ATPase data indicates that the R- and N-proteins from either the CglI or NgoAVII systems act together within a single protein–DNA complex. Indeed, only the R-proteins show a cognate DNA binding activity through their B3 domains (Figure 2, Supplementary Figures S11 and S12), that presumably triggers the DNA-dependent ATPase activity of the N-proteins. Moreover, a CglI complex with an R_2_N_2_ stoichiometry could be expressed and isolated (Table 1), or reconstituted *in vitro*. The maximum rate of ATP hydrolysis of the complexes was ∼176 ± 13 and ∼174 ± 25 molecules per second per monomer for N.CglI and N.NgoAVII, respectively. These values are closer to those reported for Type I restriction enzymes (e.g. EcoR124I ∼998 ATP/s/monomer at 30°C) that use ATP hydrolysis (1–1.5 ATP per bp step) for translocation on intact DNA (26,31,32) rather than to those reported for Type III enzymes (∼0.3–0.6 ATP/s/monomer), where ATP hydrolysis is used to drive a molecular switches to activate DNA sliding (33).

### DNA cleavage by the R-proteins

The R-proteins possess an N-terminal nucleolytic PLD-superfamily domain, also identified in the dimeric Type IIS REase BfiI (Supplementary Figure S2) (12,34). As observed with BfiI (34), we show here that the isolated R-proteins can dimerize (Table 1). They also specifically interact with the cognate sequence 5′-GCCGC-3′, suggesting that their B3 domains are responsible for target recognition, in analogy to BfiI (Figure 2 and Supplementary Figure S12). The crystal structure of apo-R.NgoAVII revealed that R.NgoAVII and BfiI are indeed structurally very similar proteins (see the accompanying paper) (9,10). As seen with BfiI, two PLD domains within the R.NgoAVII dimer form a single nuclease active site. The isolated B3 domains of R.NgoAVII (and BfiI) specifically interact with the recognition sequence as monomers (see the accompanying paper) (9,35). The R-proteins also hydrolyze the artificial substrate bis(*p*-nitrophenyl) phosphate, as demonstrated for BfiI. However, in contrast to BfiI, the R-proteins dimers in isolation were unable to cleave cognate DNA (Stonyte, Toliusis and Siksnys, unpublished results) (12). We show here that, in contrast to BfiI, they require interaction with the catalytically active ATPase subunits N.CglI or N.NgoAVII and ATP hydrolysis (Figure 4A and Supplementary Figure S15A). All natural deoxy- and ribonucleotides ((d)NTPs) could be utilized for DNA cleavage (data not shown). The dependence on divalent metal ions is likely due to the requirements of the N-proteins, since the nucleolytic PLD-superfamily domains of the R-proteins should be active without metal ions (12). Mutations of the conserved amino-acids residues from the HXK active site motifs within the nucleolytic PLD-superfamily domain (mutation H105A in R.CglI and H104A in R.NgoAVII) abolished DNA cleavage.

Both R-proteins cleaved DNA at fixed positions downstream of the asymmetric recognition sequence 5′-GCCGC-3′: R.CglI cuts the top and bottom DNA strands 7 and 6/7, R.NgoAVII - both strands 7 nucleotides downstream of the target, respectively (Figure 4B and Supplementary Figure S15B). The cleavage positions resemble that of BfiI, which cuts the top and bottom DNA strands 5 and 4 nucleotides downstream of its recognition sequence 5′-ACTGGG-3′, respectively (36). Thus the cleavage pattern can be said to be Type IIS-like.

### Functional complex

**Figure 5.**
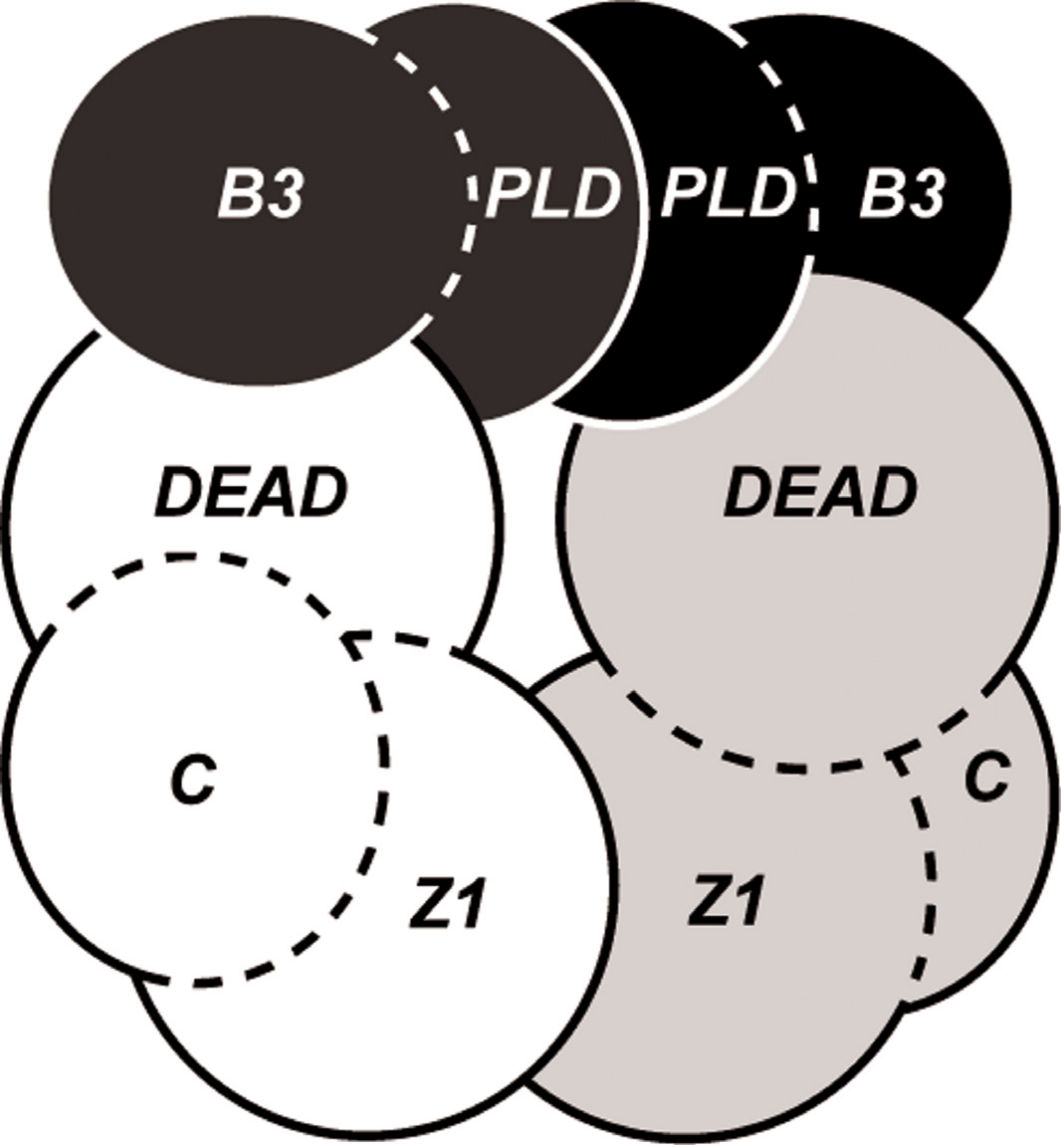
Model of the RN.CglI complex. R.CglI is composed of the PLD and B3 domains (colored black and dark gray). N.CglI contains the DEAD, Z1 and C-terminal domains (colored white and light gray).

According to the experimental data presented here, the R- and N-proteins act together as a heterotetrameric complex to hydrolyze ATP and thus cleave DNA. Based on the crystal structure of R.NgoAVII (see the accompanying paper) (9), the gel filtration data and the SAXS data, we suggest a model for the RN.CglI complex (Figure 5); although a stable NgoAVII R_2_N_2_ complex was not obtained *in vitro*, we suggest a similar complex is likely exist *in vivo*. R.CglI dimerizes through its PLD domains, to form a single nuclease active site. The B3 domains within this dimer are situated on the opposite sides of the PLD domain core and make no contacts to each other. N.CglI uses its Z1 domains for dimerization, with the DEAD domains responsible for interaction with R.CglI. On cognate DNA, an R-protein within the complex will first should bind the recognition sequence. This triggers the N-protein to hydrolyze ATP; we suggest that the ATP hydrolysis levels are consistent with translocation of the complex along DNA. DNA cleavage should proceed after translocation because the catalytically inactive R-protein stimulates the ATPase activity of the N-protein and not *vice versa*.

Interestingly, DNA cleavage occurs at fixed position near to the recognition sequence as in the case of ATP-independent BfiI. This raises the question: how can translocation away from the site then lead to cleavage close to the site? According to the crystal structure of R.NgoAVII, the R_2_N_2_ complex should contain a single nuclease active site that could produce a double-stranded DNA break through sequential strand cleavage. Instead one could speculate that two RN-complexes are required to interact and form a catalytically competent supercomplex (possibly one translocating along DNA and a second bound to the recognition sequence). In this model the two interacting R-protein dimers would each cut one strand to introduce a double-stranded DNA break near to the occupied target site. Such a mechanism is reminiscent of that suggested for the Type III restriction enzymes, although communication between the sites in the Type III case is likely by diffusion and not translocation (33,37). Experimental studies are now underway to elucidate the cleavage and possible communication mechanisms for CglI and NgoAVII endonucleases.

### Homologs of the CglI and NgoAVII RM systems

Seven more homologs of the CglI and NgoAVII RM systems were previously identified in sequenced microbial genomes (7). Two of them, the BceSIV and Bce14579I RM systems from *B. cereus* (ATCC 10987 and 14579, respectively), have been biochemically characterized (7). Analogous to the CglI and NgoAVII enzymes, BceSIV recognizes the 5′-GCWGC-3′ sequence (W stands for A and T) and acts as a complex of a PLD-superfamily nuclease and an ATPase. ATP or GTP hydrolysis was required for DNA cleavage by BceSIV. According to product analysis of partially digested plasmid using run-off sequencing it was determined that BceSIV cuts DNA on both sides of the target site in somewhat asymmetrical manner (/N9-11 GCWGC N5-7/), although this remains to be verified. According to bioinformatic analysis over 95 homologs of CglI/NgoAVII were identified in which the PLD-nuclease and ATPase genes are fused (7). We propose that the CglI/NgoAVII enzymes are prototypes of a new class of RM systems with a shared and novel catalytic mechanism.

### Comparison with Type I, II and III RM systems

The CglI and NgoAVII RM systems were previously assigned as Type II RM systems (REBASE, http://rebase.neb.com) (8). From the data presented in this paper, we can reconsider the previous assignment and compare them with the classical Type I, II and III RM systems (Table 2). The methyltransferases M.CglI and M.NgoAVII recognize a short DNA sequence (5′-GCSGC-3′) and are active separately from the REases consistent with a Type II classification (Supplementary Figure S5). They are also share sequence homology with *bona fide* Type II 5-cytosine MTases (16,17). Also consistent with the original classification, the R-proteins cleave DNA at fixed positions downstream of their asymmetric recognition sequences, similarly to Type IIS REases as exemplified by the structurally-related BfiI (Table 2) (see the accompanying paper) (9). However, DNA cleavage by CglI and NgoAVII is dependent on ATP hydrolysis performed by the helicase-like N-proteins (Table 2). The Type I and III restriction enzymes also contain SF2 helicase-like motifs and require ATP hydrolysis for DNA cleavage (Table 2) (38,39). The ATP consumption levels suggest that CglI and NgoAVII may function similarly to Type I restriction enzymes. However, their subunit composition and cleavage patterns are quite different (Table 2). Taken together, the CglI and NgoAVII REases are structurally distinctive and appear to employ a new catalytic mechanism for DNA cleavage. Further mechanistic analysis will reveal whether these enzymes can be shoehorned into one of the classical types, or whether they are indeed prototypes of an entirely distinct class of restriction-modification system.

**Table 2.** Comparison of the CglI/NgoAVII and Type I, II and III RM systems

Feature	CglI/NgoAVII	Type I	Type IIS	Type III
		e.g. EcoR124I	e.g. BfiI	e.g. EcoPI
Proteins	M (methylase)	HsdS (target recognition)	M1 (methylase)	Mod (target recognition, methylase)
	R (endonuclease)	HsdM (methylase)	M2 (methylase)	Res (helicase-endonuclease)
	N (ATPase)	HsdR (helicase-endonuclease)	R (endonuclease)	
Methyltransferase	M	M_2_S_1_, R_2_M_2_S_1_	M1, M2^b^	Mod_2_Res_1_, Mod_2_
Endonuclease	R_2_N_2_^a^	R_2_M_2_S_1_	R_2_	Mod_2_Res_1_
Nuclease domain	PLD	PD-(D/E)XK	PLD	PD-(D/E)XK
Helicase domain	SF2 (DEAD)	SF2 (DEAD)		SF2
Recognition sequence	Short, asymmetric GCCGC	Asymmetric, bipartite GAANNNNNNRTCG	Short, asymmetric ACTGGG	Short, asymmetric AGACC
DNA cleavage position	Fixed	Variable	Fixed	Fixed
Cofactors required for DNA hydrolysis	Mg^2+^, ATP	Mg^2+^, ATP, AdoMet		Mg^2+^, ATP
ATPase rate	∼170	∼998		∼0.7
	ATP/s/monomer	ATP/s/motor		ATP/s/complex
	(37ºC)	(30ºC)		(37ºC)

Data for EcoR124I was collated from (31,38). Data for EcoPI was collated from (29,38,39) and for BfiI was collated from (12,34,36).

^a^Complex stoichiometry was determined only for CglI.

^b^There are two independent BfiI methylases each of them modifying bases on the opposite strands of the asymmetric recognition site (12).

## SUPPLEMENTARY DATA

Supplementary Data are available at NAR Online.

SUPPLEMENTARY DATA
