# Supplementary material for: DNA cleavage by CgII and NgoAVII requires interaction between N- and R-proteins and extensive nucleotide hydrolysis
Source: Nucleic Acids Res. 2014 Nov 27;42(22):13887–96. doi: 10.1093/nar/gku1236 (PMC4267653; doi:10.1093/nar/gku1236)
Supplement: SUPPLEMENTARY DATA [file supp_42_22_13887__index.html]

DNA cleavage by CgII and NgoAVII requires interaction between N- and R-proteins and extensive nucleotide hydrolysis — SUPPLEMENTARY DATA 

# DNA cleavage by CgII and NgoAVII requires interaction between N- and R-proteins and extensive nucleotide hydrolysis

## SUPPLEMENTARY DATA

**Files in this Data Supplement:**

- SUPPLEMENTARY DATA
