# Supplementary material for: DNA cleavage by CgII and NgoAVII requires interaction between N- and R-proteins and extensive nucleotide hydrolysis
Source: Nucleic Acids Res. 2014 Nov 27;42(22):13887–96. doi: 10.1093/nar/gku1236 (PMC4267653; doi:10.1093/nar/gku1236)
Supplement: SUPPLEMENTARY DATA [file supp_gku1236_nar-02802-z-2014-File007.pdf]

Supplementary data to:

DNA cleavage by CglI and NgoAVII requires interaction between N- and R-proteins and extensive nucleotide hydrolysis

Mindaugas Zaremba[1,*], Paulius Toliusis[1], Rokas Grigaitis[1], Elena Manakova[1], Arunas Silanskas[1], Giedre Tamulaitiene[1], Mark D. Szczelkun[2] and Virginijus Siksnys[1,*]

[1] Department of Protein–DNA Interactions, Institute of Biotechnology, Vilnius University, Graiciuno 8, LT-02241, Vilnius, Lithuania
[2] DNA–Protein Interactions Unit, School of Biochemistry, Medical Sciences Building, University of Bristol, Bristol BS8 1TD, U.K.

* To whom correspondence should be addressed. Tel: +370-5-2602111; Fax: +370-5-2602116; Email: zare@ibt.lt. Correspondence may also be addressed to Virginijus Siksnys. Tel: +370-5-2602108; Fax: +370-5-2602116; Email: siksnys@ibt.lt.

The authors wish it to be known that, in their opinion, the first two authors should be regarded as joint First Authors.

SUPPLEMENTARY DATA

Supplementary text

DNA substrates for ATP hydrolysis studies

In these experiments the following DNA substrates were used: (i) a linear double-stranded phage λ DNA (48.5 kb length) containing 181 recognition sites for CglI/NgoAVII; (ii) a circular supercoiled double-stranded pBR322 plasmid (4.4-5.5 kb) containing 21 unmethylated or 22 methylated CglI or NgoAVII recognition sites, respectively (the methylated pBR322 contains the gene of the M.NgoAVII methyltransferase, which methylates CglI/NgoAVII recognition sequences); (iii) a linear double-stranded cognate and non-cognate DNA fragments (281 bp) with and without the CglI/NgoAVII target, respectively; (iv) a 16 bp cognate and non-cognate oligoduplexes with or without the CglI/NgoAVII target, respectively; (v) a circular single-stranded phage M13mp18 DNA. The same DNA concentration (0.01 μg/μl) was used in all reactions.

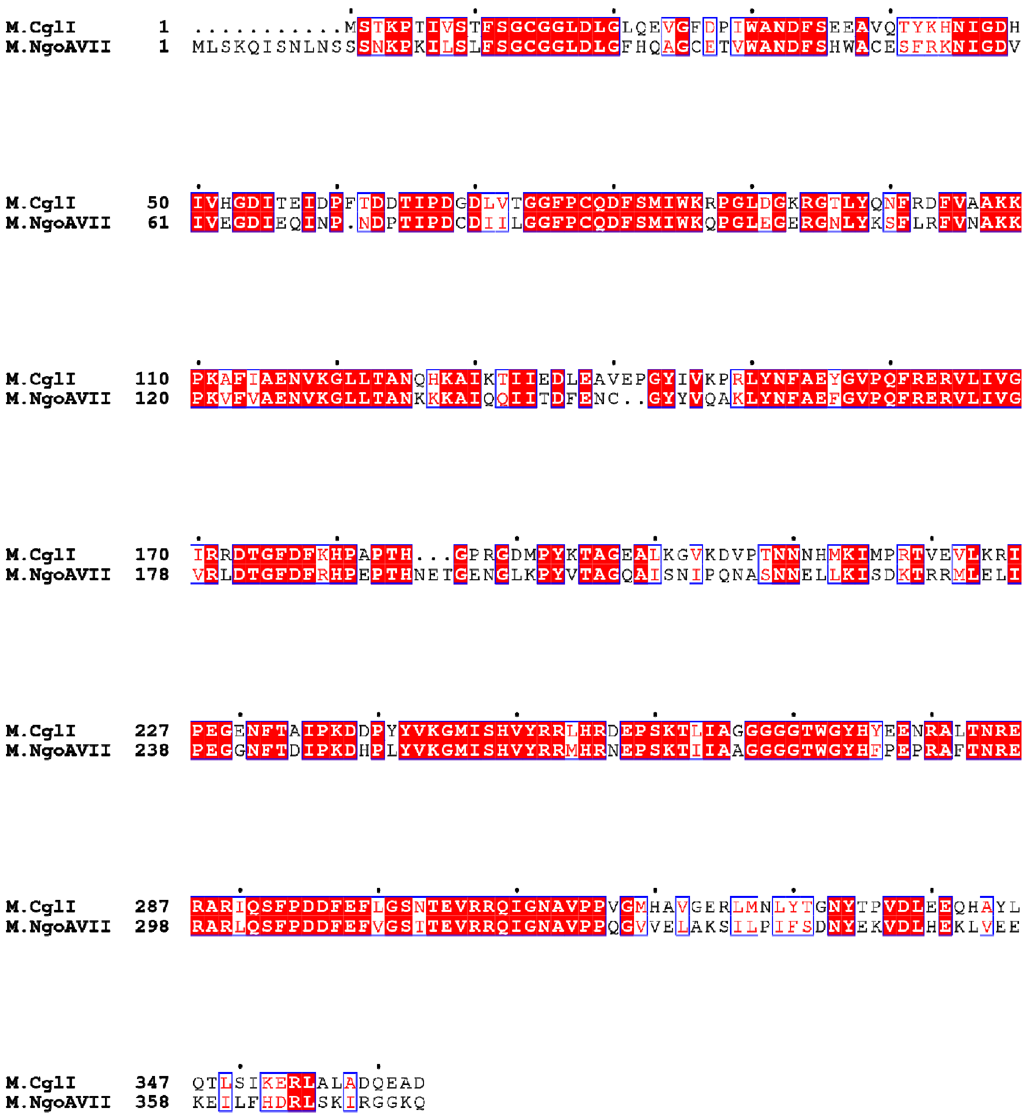

Supplementary Figure S1. Amino acid sequence alignment of M.CglI and M.NgoAVII. The alignment was produced with MULTALIN (40) and rendered with ESPRIPT (41).

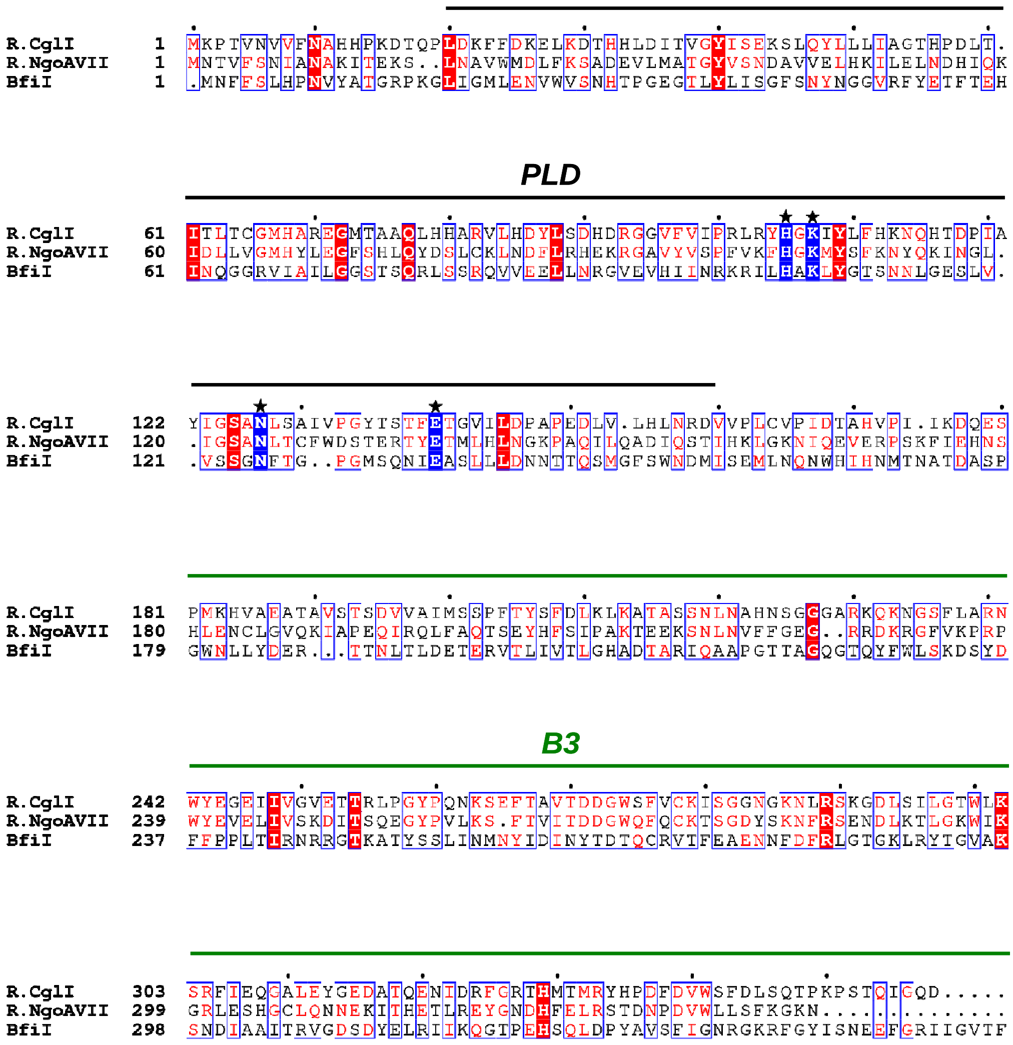

Supplementary Figure S2. Amino acid sequence alignment of R.CglI, R.NgoAVII and BfiI. The PLD-superfamily nucleolytic and B3-like DNA binding domains are marked by black and green strips, respectively. The putative catalytic residues are marked by stars; the H105 (R.CglI) and H104 (R.NgoAVII) residues were subjected to mutagenesis. The protein sequence alignment was produced with MULTALIN (40) and rendered with ESPRIPT (41). Location of conserved domain footprints and functional sites was identified with CDD (42).

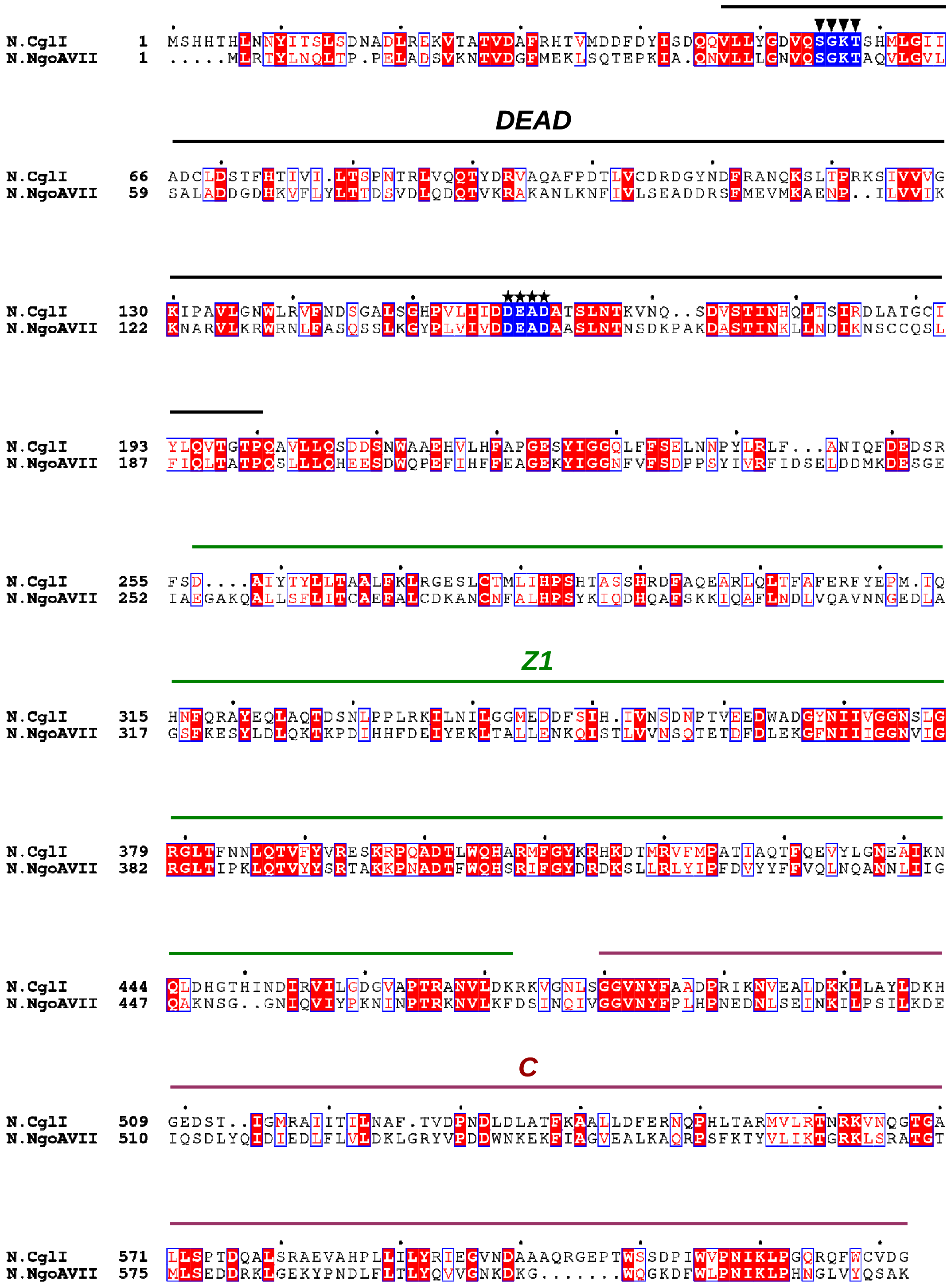

Supplementary Figure S3. Amino acid sequence alignment of N.CglI and N.NgoAVII. The DEAD-superfamily, Z1-superfamily and C-terminal domains are marked by black, green and brown strips, respectively. The putative residues responsible for ATP and Mg^{2+} binding are indicated by downward triangles and stars, respectively. The D158/E159 (R.CglI) and D150/E151 (R.NgoAVII) residues from the Mg^{2+} binding site were subjected to mutagenesis. The protein sequence alignment was produced with MULTALIN (40) and rendered with ESPRIPT (41). Location of conserved domain footprints and functional sites was identified with CDD (42).

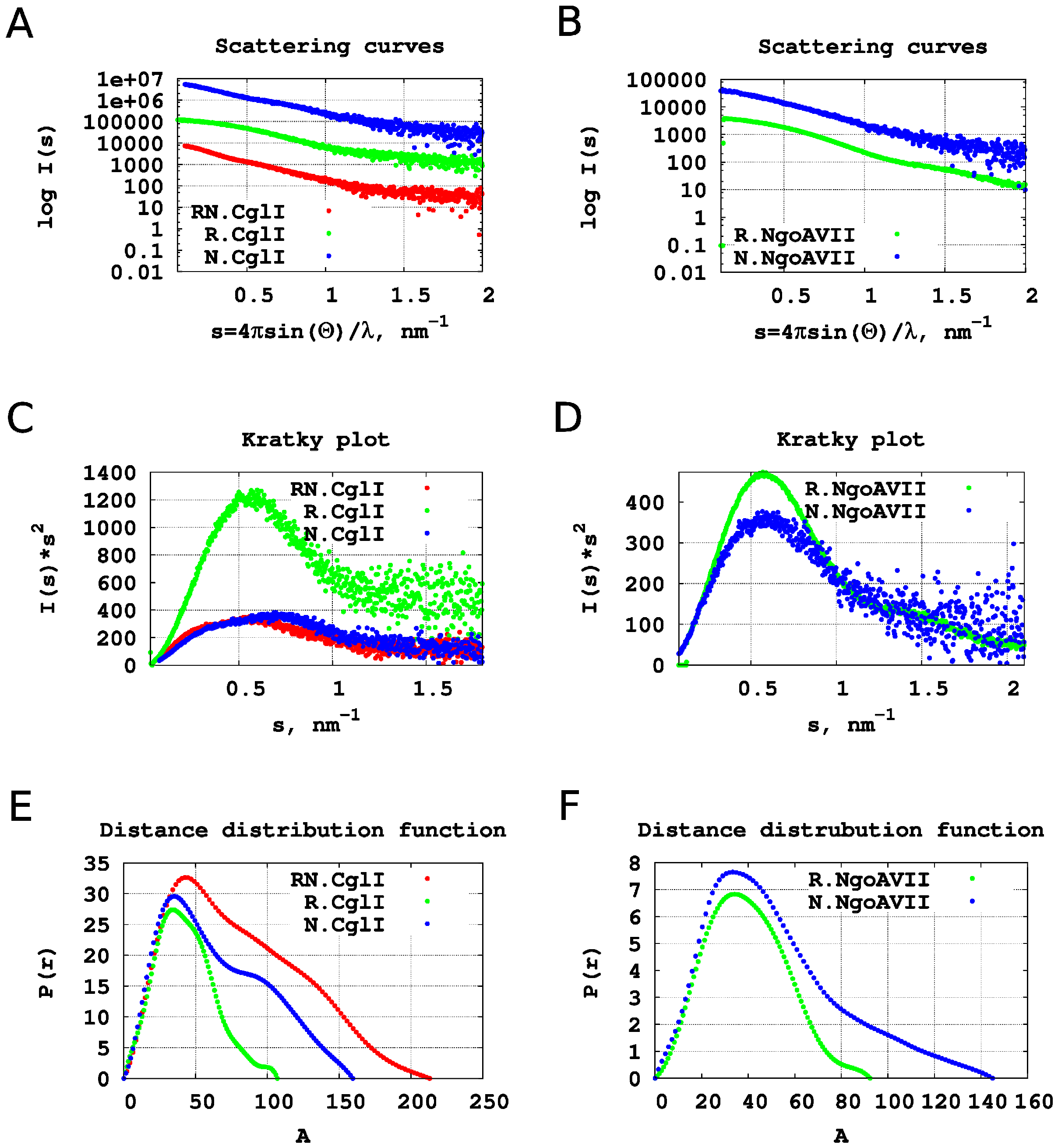

Supplementary Figure S4. SAXS data of CglI and NgoAVII. A and B, scattering profiles of CglI and NgoAVII, respectively, shown as a logarithmic plot of scattering intensity I(s) vs s = 4π sin(θ)/λ, where 2θ is a scattering angle and λ is X-ray wavelength. B and D, Kratky plot, I(s)*s^2 vs s. E and F, Distance distribution functions.

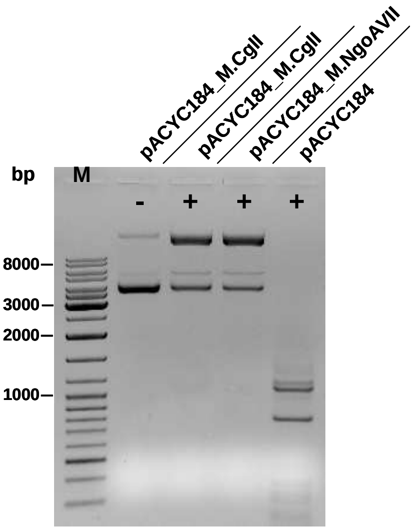

Supplementary Figure S5. Resistance of DNA to TauI cleavage. A purified plasmid pACYC184 containing the gene of M.CglI or M.NgoAVII was resistant to the cleavage by the restriction endonuclease TauI that recognizes the same 5′-GCSGC-3′ DNA sequence. Addition of FastDigest TauI (Thermo Fisher Scientific, Vilnius) is indicated by “+”.

A

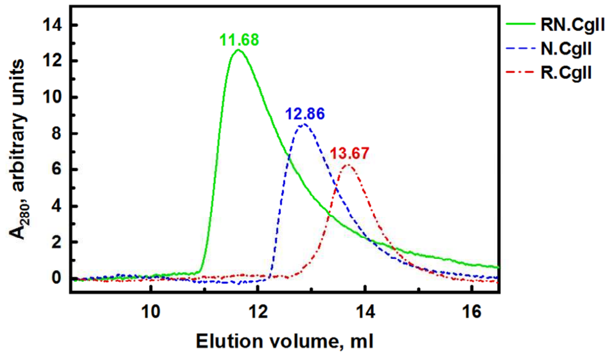

B

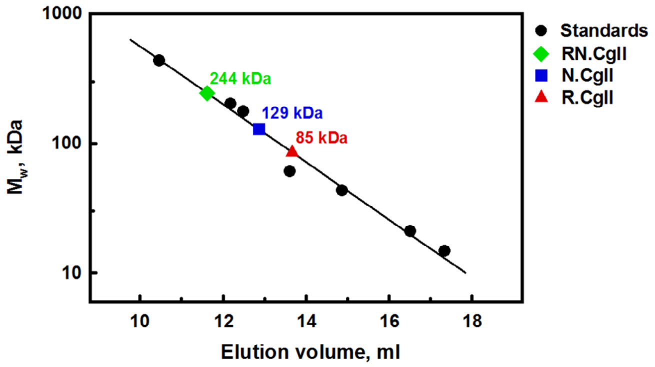

Supplementary Figure S6. Gel filtration of the CglI proteins. (A) Elution profiles of the CglI proteins. Gel filtration of the individual R.CglI, N.CglI and their RN.CglI complex was carried out as described in 'Materials and Methods'. (B) The apparent molecular weights of the CglI proteins were evaluated from the elution volume using a series of standards (Gel Filtration Calibration Kit from GE).

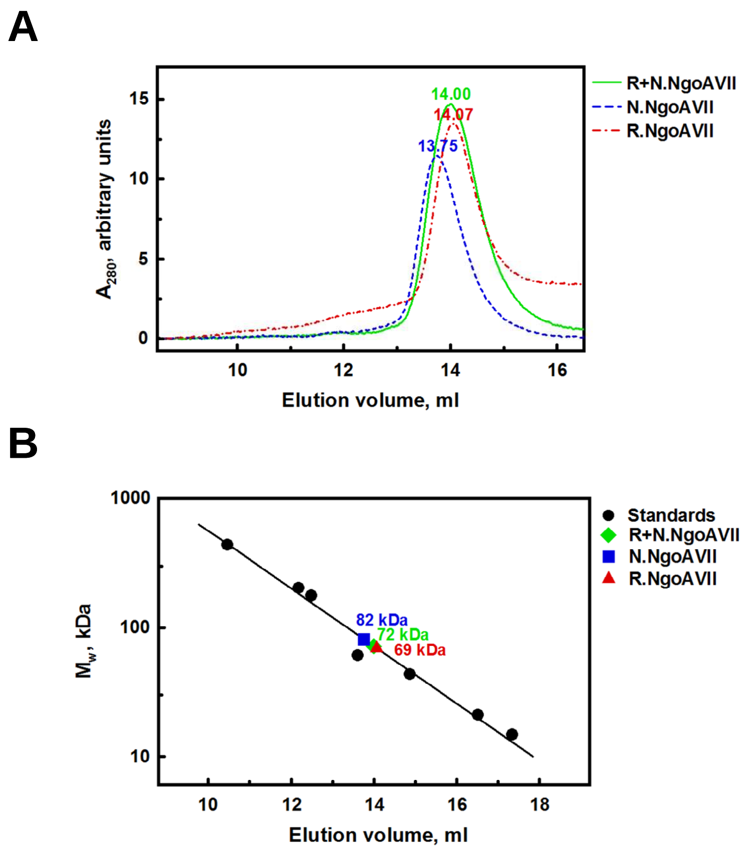

Supplementary Figure S7. Gel filtration of the NgoAVII proteins. (A) Elution profiles of the R.NgoAVII proteins. Gel filtration of the individual R.NgoAVII, N.NgoAVII and their mix (R+N.NgoAVII) was carried out as described in 'Materials and Methods'. (B) The apparent molecular weights of the NgoAVII proteins were evaluated from the elution volume using a series of standards (Gel Filtration Calibration Kit from GE).

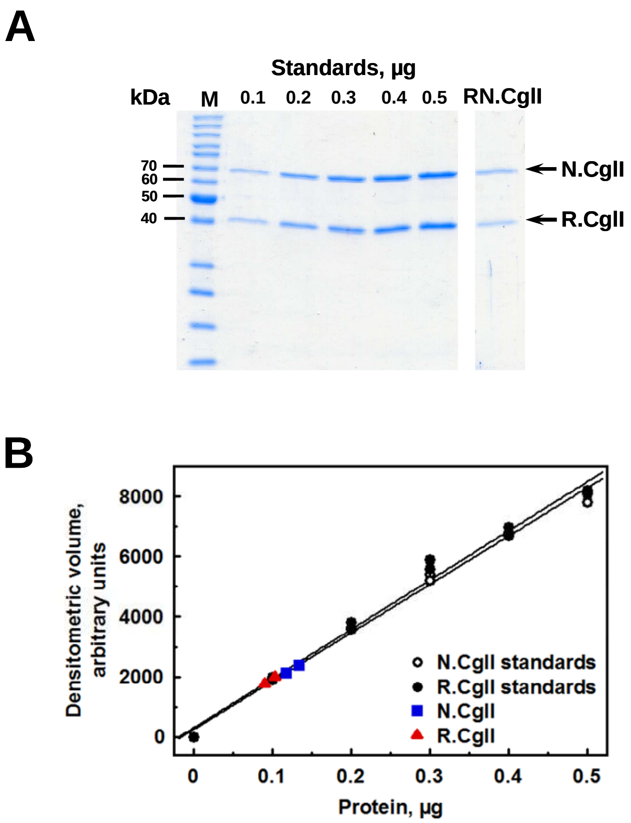

Supplementary Figure S8. Densitometric analysis of the RN.CglI complex. (A) SDS polyacrylamide gel with the R.CglI and N.CglI protein standards and the RN.CglI complex. (B) The amounts of the R.CglI and N.CglI proteins from the RN.CglI complex were evaluated from the densitometric volumes of the R.CglI and N.CglI standards. SDS/PAGE gels were stained with PageBlue Protein Staining Solution (Thermo Fisher Scientific), scanned with the EPSON PERFECTION V300 PHOTO scanner and analysed using ImageJ software (43).

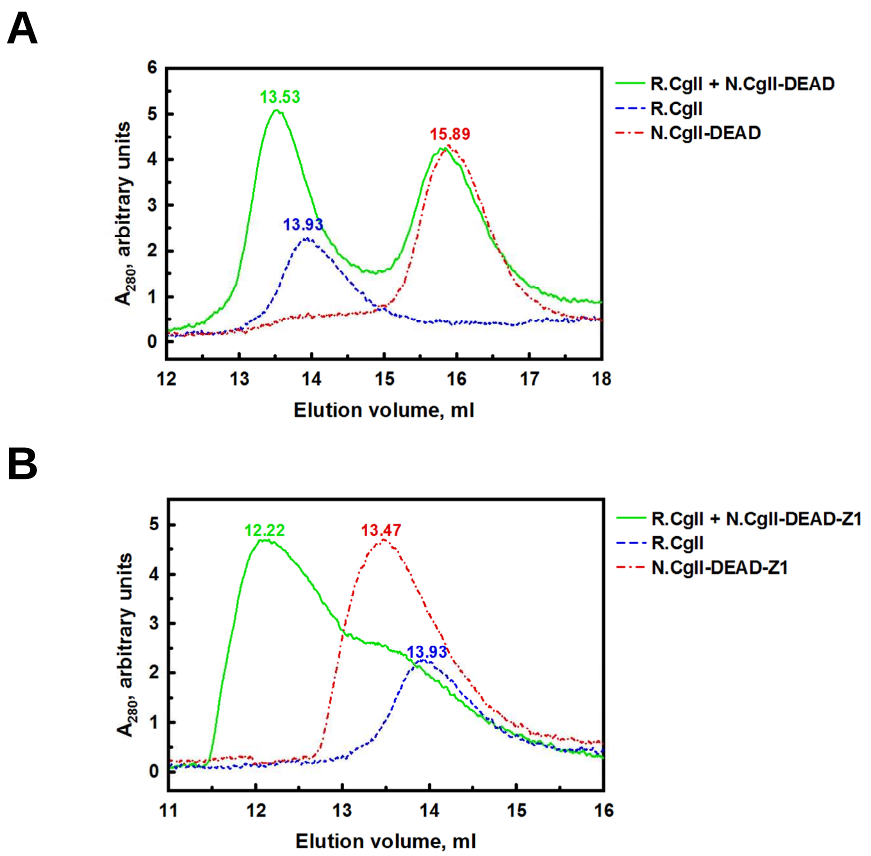

Supplementary Figure S9. Gel filtration of the R.CglI and N.CglI domains. Gel filtration of the individual R.CglI, N.CglI-DEAD, N.CglI-DEAD-Z1 and their mixtures (A and B) was carried out as described in ‘Materials and Methods’.

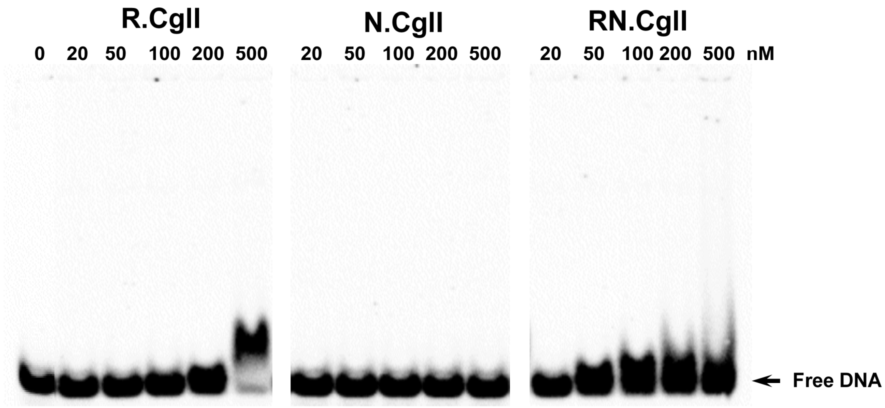

Supplementary Figure S10. Non-cognate DNA binding by CglI proteins. The reactions contained 1 nM of the ^{33}P-labeled non-cognate oligoduplex and the protein at the concentrations indicated above each lane. After 15 min at room temperature, the samples were subjected to PAGE for 3 h and analysed as described in ‘Materials and Methods’.

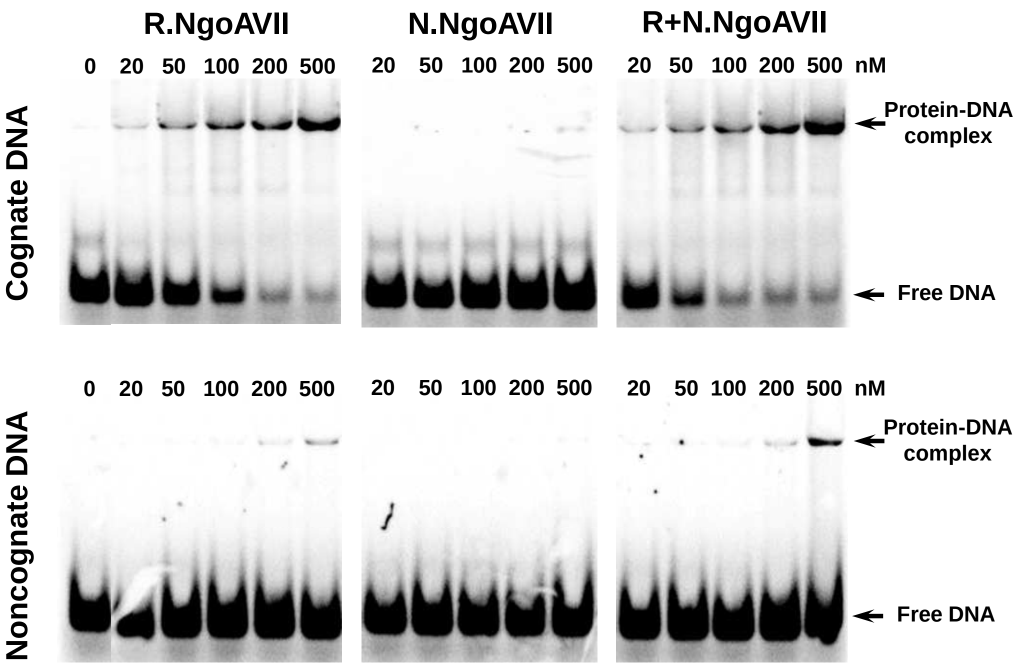

Supplementary Figure S11. DNA binding by NgoAVII proteins. The reactions contained 1 nM of the ^{33}P-labeled cognate and non-cognate oligoduplex and the protein at the concentrations indicated above each lane. After 15 min at room temperature, the samples were subjected to PAGE for 3 h and analysed as described in 'Materials and Methods'.

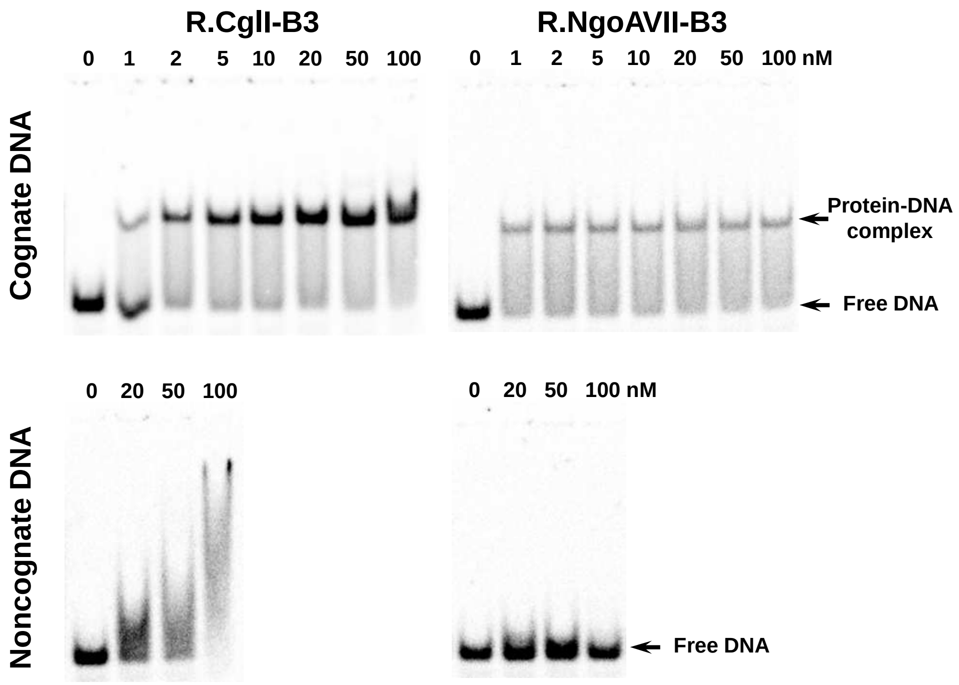

Supplementary Figure S12. DNA binding by R.CglI-B3 and R.NgoAVII-B3 domains. The reactions contained 1 nM of the ^{33}P-labeled cognate and non-cognate oligoduplex and the protein at the concentrations indicated above each lane. After 15 min at room temperature, the samples were subjected to PAGE for 3 h and analysed as described in 'Materials and Methods'. The R.CglI-B3 domain forms an unstable protein-DNA complex with the non-cognate DNA at high protein concentrations.

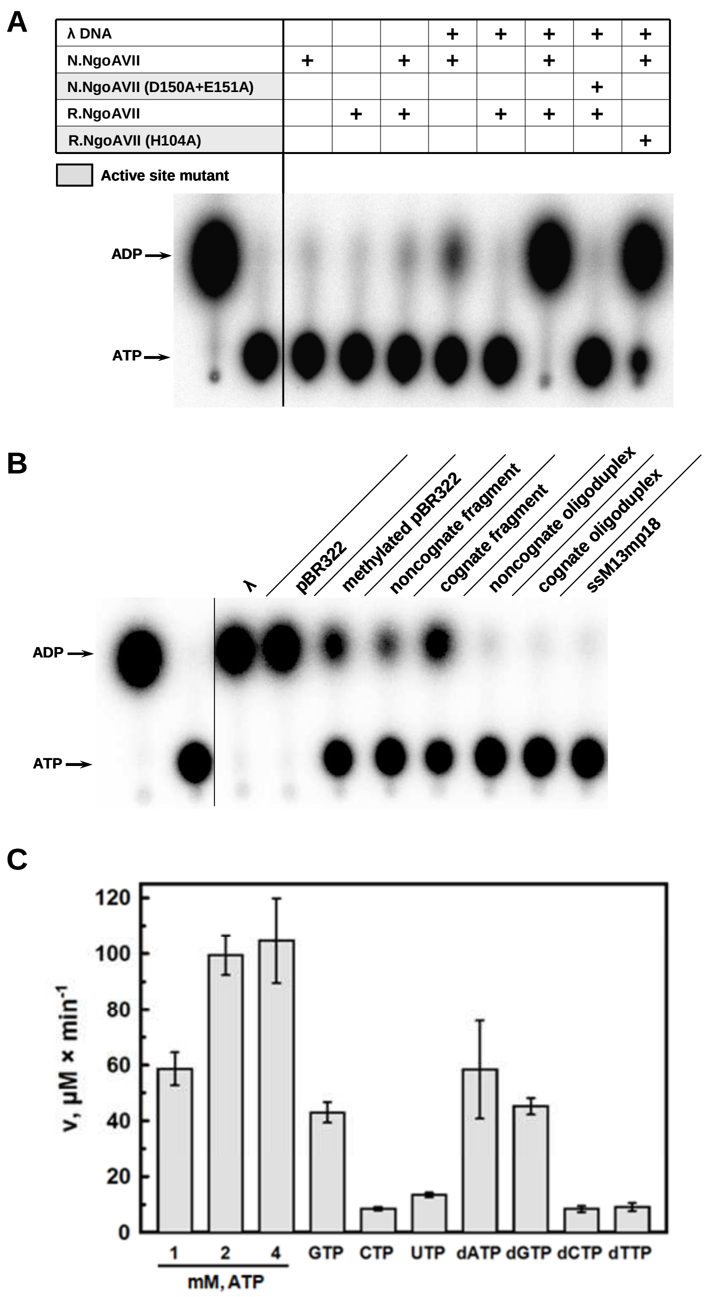

Supplementary Figure S13. N.NgoAVII ATPase activity. (A) Radioactive ATPase assay. ATPase reactions contained 50 μM [α^{32}P]ATP, 0.01 μg/μl phage λ DNA, 100 nM N.NgoAVII or R.NgoAVII and were conducted as described in 'Materials and Methods'. Reaction products were separated using thin-layer chromatography and visualized using a phosphoimager. (B) Dependence of NgoAVII ATPase activity on different DNAs. Reactions were performed as in (A) using 40 nM N.NgoAVII or R.NgoAVII and 0.01 μg/μl DNA (see details in the text). (C) (d)NTP hydrolysis rates. Reactions contained 0.02 μg/μl phage λ DNA, 10 nM N.NgoAVII, 200 nM R.NgoAVII, 1-4 mM ATP or 1 mM (d)NTP, as indicated, and were conducted as described in 'Materials and Methods'. The malachite green assay was used to measure ATP hydrolysis through the detection of liberated-free phosphate from ATP.

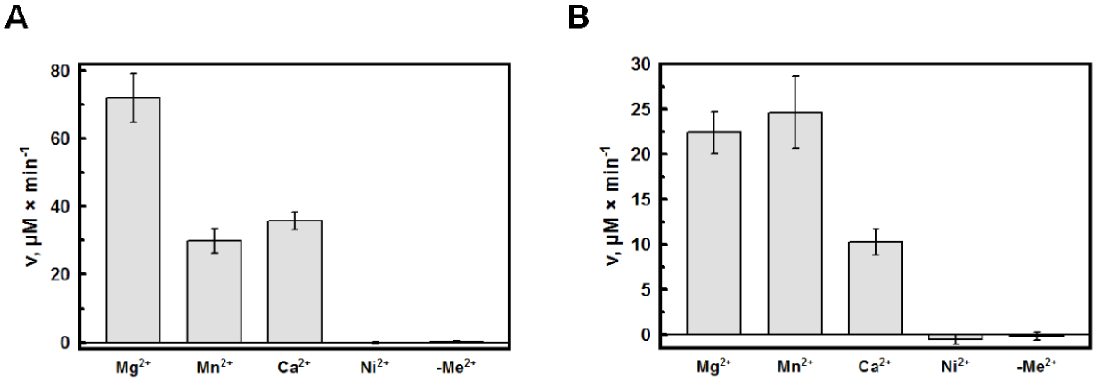

Supplementary Figure S14. Dependence of N.CglI (A) and N.NgoAVII (B) ATPase activity on different metal ions. Reactions contained 0.02 µg/µl phage λ DNA, 10 nM N.CglI or N.NgoAVII, 200 nM R.CglI or R.NgoAVII, 1 mM ATP, 10 mM dvivalent metal ions (Mg-acetate, $MnCl_2$, $CaCl_2$, $NiCl_2$) as indicated, and were conducted as described in 'Materials and Methods'. The malachite green assay was used to measure ATP hydrolysis through the detection of liberated-free phosphate from ATP.

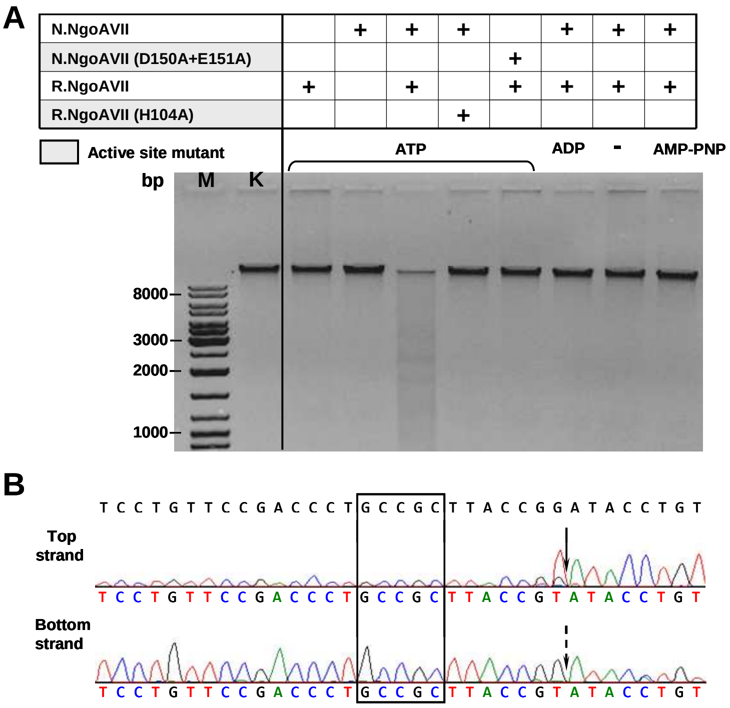

Supplementary Figure S15. R.NgoAVII nuclease activity and cleavage site mapping. (A) Bacteriophage λ DNA cleavage by R.NgoAVII. Reactions contained 0.01 μg/μl bacteriophage λ DNA, 100 nM R.NgoAVII or N.NgoAVII, 2 mM ATP, ADP or AMP-PNP (as indicated above lanes) and were conducted as described in 'Materials and Methods'. (B) Run-off sequencing to determine the cleavage position of R.NgoAVII. The recognition sequence 5′-GCCGC-3′ is indicated by the rectangle, with the cleavage sites are indicated by arrows.

Supplementary Table S1. Expression and purification details of CglI and NgoAVII proteins.

Protein /complex	Length (without tag), a.a.	Affinity tag[a], terminus	Molecular weight, Da[b]	Extinction coefficient, $M^{-1}cm^{-1}$[b]	Expression vector[c], antibiotic resistance	*E. coli* strain[d], antibiotic resistance	Expression temperature, duration, inducer[e]	Purification columns[f]	Storage buffer[g]
R.CglI	358	StrepII, N	41242.5	45380	pBAD24, Ap	ER2267, Kn	16ºC, overnight, 0.2% (w/v) L(+)-arabinose	StrepTrap HP, MonoQ	2
N.CglI	632	His_6, C	71933.9	66810	pBAD24, Ap	ER2267, Kn	16ºC, overnight, 0.2% (w/v) L(+)-arabinose	HisTrap HP, HiPrep desalting	2
R.CglI<br>+<br>N.CglI	358<br><br>632	StrepII, N<br><br>His_6, C	41242.5<br><br>71933.9	45380<br><br>66810	pETDuet-1, Ap	ER2566, -	16ºC, overnight, 1 mM IPTG	HisTrap HP, StrepTrap HP, Superdex™ 200 10/300 GL	2
R.NgoAVII	345	His_6, N	42000.5	50880	pET15b, Ap	ER2566, -	16ºC, overnight, 0.4 mM IPTG	HisTrap HP, HiPrep desalting	1
N.NgoAVII	629	His_6, C	72223.1	65780	pBAD24, Ap	ER2267, Kn	37ºC, 3h, 0.2% (w/v) L(+)-arabinose	HisTrap HP, HiTrap Heparin, HiTrap Q FF	2
R.NgoAVII<br>+<br>N.NgoAVII	345<br><br>629	StrepII, N<br><br>His_6, C	42000.5<br><br>72223.1	50880<br><br>65780	pBAD24, Ap	ER2267, Kn	37ºC, 3 h, 0.2% (w/v) L(+)-arabinose	HisTrap HP, StrepTrap HP	-

[a]Affinity tags were not removed before the experiments.
[b]Molecular weights and extinction coefficients were calculated using the ProtParam tool, http://web.expasy.org/protparam/.
[c]pBAD24 was from Invitrogen, pETDuet-1 and pET15b were from Novagen.
[d]*E. coli* strain contained the pBsp6I plasmid (chloramphenicol resistance) with a gene of the Bsp6I methyltransferase that modifies the CglI/NgoAVII recognition sequence.
[e]Initially cells were grown in LB broth supplemented with proper antibiotics (ampicillin (100 µg/ml), kanamycin (25 µg/ml), chloramphenicol (30 µg/ml)) at 37℃ to OD_{600} of ~0.5-0.6, then expression was induced as indicated.
[f]All columns were from GE Healthcare. Columns were used in the sequence as indicated. The following buffers were used: Buffer 1 (20 mM Tris-HCl (pH 8.0 at 25°C), 1 M NaCl, 25 mM imidazole, 5 mM 2-mercaptoethanol) for HisTrap HP; Buffer 2 (20 mM Tris-HCl (pH 8.0 at 25°C), 1 M NaCl, 5 mM 2-mercaptoethanol) for HiPrep desalting, MonoQ, HiTrap Heparin and HiTrap Q FF; Buffer 3 (20 mM Tris-HCl (pH 8.0 at 25°C), 100 mM NaCl, 5 mM 2-mercaptoethanol) for HiPrep desalting, MonoQ and Heparin; Buffer 4 (20 mM Tris-HCl (pH 8.0 at 25°C), 50 mM NaCl, 5 mM 2-mercaptoethanol) for HiTrap Q FF and MonoQ; Buffer 5 (20 mM Tris-HCl (pH 8.0 at 25°C), 300 mM NaCl, 5 mM 2-mercaptoethanol) for StrepTrap HP; Buffer 6 (20 mM Tris-HCl (pH 8.0 at 25°C), 500 mM NaCl, 5% (v/v) glycerol) for HisTrap HP and Superdex™ 200 10/300 GL; Buffer 7 (20 mM Tris-HCl (pH 8.0 at 25°C), 1 M NaCl, 500 mM imidazole, 5 mM 2-mercaptoethanol) for HisTrap HP; Buffer 8 (20 mM Tris-HCl (pH 8.0 at 25°C), 1 M NaCl, 2.5 mM desthiobiotin, 5 mM 2-mercaptoethanol) for StrepTrap HP; Buffer 9 (20 mM Tris-HCl (pH 8.0 at 25°C), 500 mM NaCl, 500 mM imidazole, 5% (v/v) glycerol) for HisTrap HP. All purifications were performed in accordance with manufacturer's instructions.
[g]Storage buffer 1: 20 mM Tris-HCl (pH 8.0 at 25℃), 200 mM KCl, 2 mM DTT, 0.1 mM EDTA, 50% (v/v) glycerol; Storage buffer 2: 20 mM Tris-HCl (pH 8.0 at 25°C), 400 mM KCl, 2 mM DTT, 0.1 mM EDTA, 50% (v/v) glycerol.

Supplementary Table S2. Expression and purification details of CgII and NgoAVII protein domains.

Protein/ domain	Length (without tag), a.a., position	Affinity tag[a], terminus	Molecular weight, Da[b]	Extinction coefficient, $M^{-1}cm^{-1}$[b]	Expression vector[c], antibiotic resistance	*E. coli* strain, antibiotic resistance	Expression temperature, duration, inducer[e]	Purification columns[f]	Storage buffer[g]
R.CgII PLD[g]	178, 1-178	StrepII, N	21771.8	15930	pETDuet, Ap	BL-21 (DE3), -	16ºC, overnight, 1 mM IPTG	HisTrap HP, HiPrep desalting	2
R.CgII B3	180, 179-358	His_6, C	20918.1	29450	pLATE31, Ap	ER2566, -	16ºC, overnight, 1 mM IPTG	HisTrap HP	1
N.CgII DEAD	229, 1-229	His_6, N	28369.4	21430	pLATE51, Ap	BL-21 (DE3), -	37ºC, 4h, 1 mM IPTG	HisTrap HP, HiTrap Q FF	2
N.CgII DEAD-Z1	463, 1-463	His_6, N	55339.8	45840	pLATE51, Ap	BL-21 (DE3), -	37ºC, 4h, 1 mM IPTG	HisTrap HP, Superdex 200 GL	2
N.CgII Z1	234, 230-463	His_6, C	28056.5	24410	pLATE31, Ap	BL-21 (DE3), -	16ºC, overnight, 1 mM IPTG	HisTrap HP, HiPrep desalting	2
N.CgII Z1-C	403, 230-632	His_6, C	46723.8	45380	pLATE31, Ap	BL-21 (DE3), -	16ºC, overnight, 1 mM IPTG	HisTrap HP, Superdex 200 GL	2
N.CgII C	169, 464-632	His_6, C	19753.5	20970	pLATE31, Ap	BL-21 (DE3), -	16ºC, overnight, 1 mM IPTG	HisTrap HP, HiPrep desalting	2
R.NgoAVII B3	167, 179-345	His_6, C	20631.0	29450	pLATE31, Ap	ER2566, -	16ºC, overnight, 1 mM IPTG	HisTrap HP	1
N.NgoAVII DEAD	229, 1-229	His_6, N	28691.4	16960	pLATE51, Ap	ER2566, -	16ºC, overnight, 1 mM IPTG	HisTrap HP, HiTrap SP FF, HiTrap Q FF, Superdex 200 GL	1
N.NgoAVII DEAD-Z1	463, 1-463	His_6, N	55232.5	38850	pLATE51, Ap	ER2566, -	16ºC, overnight, 1 mM IPTG	HisTrap HP, HiTrap Q FF, Superdex 200 GL	1
N.NgoAVII Z1	234, 230-463	His_6, C	27627.2	21890	pLATE31, Ap	BL-21 (DE3), -	37ºC, 4h, 1 mM IPTG	HisTrap HP, HiPrep desalting, HiTrap Heparin	1
N.NgoAVII Z1-C	400, 230-629	His_6, C	46691.1	48820	pLATE31, Ap	BL-21 (DE3), -	37ºC, 4h, 1 mM IPTG	HisTrap HP, HiPrep desalting, HiTrap Heparin	1
N.NgoAVII C	166, 464-629	His_6, C	20150.0	26930	pLATE31, Ap	BL-21 (DE3), -	37ºC, 4h, 1 mM IPTG	HisTrap HP, HiTrap Heparin, HiPrep desalting	1

[a]Affinity tags were not removed before the experiments.
[b]Molecular weights and extinction coefficients were calculated using the ProtParam tool, http://web.expasy.org/protparam/.
[c]pLATE31 and pLATE51 were from Thermo Fisher Scientific (Vilnius).
[d]*E. coli* strain contained the plasmid (chloramphenicol resistance) with a gene of the CgII methyltransferase.

[e]Initially cells were grown in LB broth supplemented with proper antibiotics (ampicillin (100 µg/ml), chloramphenicol (30 µg/ml)) at 37℃ to OD_{600} of ~0.5-0.6, then expression was induced as indicated.
[f]Purifications were performed as described in Supplementary Table S1.
[g]Storage buffers described in Supplementary Table S1.
[h]The R.CglI-PLD domain contains the active site mutation H105A.

Supplementary Table S3. Structural parameters of CglI and NgoAVII calculated from SAXS data.

	RN.CglI	R.CglI	N.CglI	R.NgoAVII	N.NgoAVII
Concentration range, mg/ml	1.16-3.56	0.3-3.86	0.82-1.51	2.75-11.6	1.14-3.58
Concentration, mg/ml	1.16	1.41	1.12	merged data	merged data
Guinier range, first point-last point (s range, Å^{-1}) as calculated by AUTORG	12 to 53 (0.0105 to 0.0214)	32 to 117 (0.0111 to 0.0335)	19 to 66 (0.0126 to 0.0249)	34 to 131 (0.0164 to 0.0421)	32 to 79 (0.0158 to 0.0283)
P(r) calculation range, Å^{-1}	0.0098 - 0.2006	0.0062 - 0.2512	0.0128 - 0.2005	0.0168 - 0.2597	0.0129 - 0.3334
Real space R_g, calculated by GNOM, Å	64.5 ± 0.3	33.5 ± 0.1	51.8 ± 0.2	30.2 ± 0.1	41.4 ± 0.5
D_{max}, as parameter for GNOM, Å	213.0	107.0	159.5	92.3	145.0
D_{max}, calculated by DATGNOM, Å	211.7	104.8	167.0	-	-
Porod volume estimated by DATPOROD, Å^3	379348	148037	195997	128733	185672
Excluded volume of DAMMIN models, Å^3 (10 models averaged)	449340 ± 3076	154660 ± 1435	241740 ± 2482	142990 ± 626	170910 ± 1203
Software used for data processing	PRIMUS, GNOM, DATPOROD, DATGNOM, AUTORG (http://www.embl-hamburg.de/biosaxs/software.html)				

Supplementary references

40. Corpet, F. (1988) Multiple sequence alignment with hierarchical clustering. Nucleic Acids Res., 16, 10881-10890.

41. Gouet, P., Robert, X. and Courcelle, E. (2003) ESPript/ENDscript: Extracting and rendering sequence and 3D information from atomic structures of proteins. Nucleic Acids Res., 31, 3320-3323.

42. Marchler-Bauer, A., Zheng, C., Chitsaz, F., Derbyshire, M.K., Geer, L.Y., Geer, R.C., Gonzales, N.R., Gwadz, M., Hurwitz, D.I., Lanczycki, C.J. *et al.* (2013) CDD: conserved domains and protein three-dimensional structure. Nucleic Acids Res., 41, D348-352.

43. Schneider, C.A., Rasband, W.S. and Eliceiri, K.W. (2012) NIH Image to ImageJ: 25 years of image analysis. Nat. Methods, 9, 671-675.
